# Anti-biofilm Properties of Bacterial Di-Rhamnolipids and Their Semi-Synthetic Amide Derivatives

**DOI:** 10.3389/fmicb.2017.02454

**Published:** 2017-12-08

**Authors:** Ivana Aleksic, Milos Petkovic, Milos Jovanovic, Dusan Milivojevic, Branka Vasiljevic, Jasmina Nikodinovic-Runic, Lidija Senerovic

**Affiliations:** ^1^Institute of Molecular Genetics and Genetic Engineering, University of Belgrade, Belgrade, Serbia; ^2^Department of Organic Chemistry, Faculty of Pharmacy, University of Belgrade, Belgrade, Serbia

**Keywords:** rhamnolipids, di-rhamnolipids, biofilms, cell adhesion, amide derivative

## Abstract

A new strain, namely *Lysinibacillus* sp. BV152.1 was isolated from the rhizosphere of ground ivy (*Glechoma hederacea* L.) producing metabolites with potent ability to inhibit biofilm formation of an important human pathogens *Pseudomonas aeruginosa* PAO1, *Staphylococcus aureus*, and *Serratia marcescens*. Structural characterization revealed di-rhamnolipids mixture containing rhamnose (Rha)-Rha-C10-C10, Rha-Rha-C8-C10, and Rha-Rha-C10-C12 in the ratio 7:2:1 as the active principle. Purified di-rhamnolipids, as well as commercially available di-rhamnolipids (Rha-Rha-C10-C10, 93%) were used as the substrate for the chemical derivatization for the first time, yielding three semi-synthetic amide derivatives, benzyl-, piperidine-, and morpholine. A comparative study of the anti-biofilm, antibacterial and cytotoxic properties revealed that di-Rha from *Lysinibacillus* sp. BV152.1 were more potent in biofilm inhibition, both cell adhesion and biofilm maturation, than commercial di-rhamnolipids inhibiting 50% of *P. aeruginosa* PAO1 biofilm formation at 50 μg mL^-1^ and 75 μg mL^-1^, respectively. None of the di-rhamnolipids exhibited antimicrobial properties at concentrations of up to 500 μg mL^-1^. Amide derivatization improved inhibition of biofilm formation and dispersion activities of di-rhamnolipids from both sources, with morpholine derivative being the most active causing more than 80% biofilm inhibition at concentrations 100 μg mL^-1^. Semi-synthetic amide derivatives showed increased antibacterial activity against *S. aureus*, and also showed higher cytotoxicity. Therefore, described di-rhamnolipids are potent anti-biofilm agents and the described approach can be seen as viable approach in reaching new rhamnolipid based derivatives with tailored biological properties.

## Introduction

Microbial biofilms are prevalent in nature, especially in medical, industrial and environmental settings where they cause undesirable effects due to their pathogenicity and resistance toward antibiotics or biofouling technologies (Berlanga and Guerrero, 2016; Holscher and Kovacs, 2017). Biofilms play an important role in virulence of many pathogenic bacteria including *Pseudomonas aeruginosa, Staphylococcus aureus*, and *Serratia marcescens* (Costerton et al., 1999; Singh et al., 2000; Bryers, 2008). They can be formed in the patients’ tissues or on the surface of medical devices associated with the human body such as catheters, naso-laryngeal tubes, or stents (Bryers, 2008). Various strategies have been developed and employed for the efficient biofilm control (Coughlan et al., 2016; Rowson and Townsend, 2016), however, new, natural, and effective anti-biofilm agents are still highly sought after. Application of microbial biosurfactants in anti-biofilm and antibiofouling efforts emerged as an attractive and one of the viable strategies in recent years (Vatsa et al., 2010; Varjani and Upasani, 2017).

Biotechnological interest in biosurfactants has grown exponentially since the 1980s when these molecules were found useful in oil recovery and bioremediation (Abdel-Mawgoud et al., 2010; Muller et al., 2012; Dobler et al., 2016). Rhamnolipids as a group of anionic microbial glycolipids, consisting of L-(+)-rhamnose and β-hydroxyalkanoic acids, have been considered in a wide range of applications such as health care, cosmetics, pharmaceutical processes, food and beverage processing, detergents and polymer industry, cryo-protectants, biofuels, microbial fuel cells, and bioremediation, due to their unique characteristics such as low toxicity, surface activities, sustainability, and biodegradability (Li, 2017; Varjani and Upasani, 2017).

Rhamnolipids were firstly described as secondary metabolites of *P. aeruginosa* in 1949 and were found essential for the growth of this bacterium on hydrophobic carbon sources (Muller et al., 2011; Gong et al., 2015), but are also critical for forming structured biofilms with pore and channels in this organism (Davey et al., 2003). So far, over 60 rhamnolipid congeners and homologs have been described, including mono- and di-rhamnolipids, containing mostly two, one, or even three molecules of β-hydroxyalkanoic acids with varying carbon chain length (Abdel-Mawgoud et al., 2010). Rhamnolipids reached the market as an active ingredient of Zonix TM fungicide (Jeneil Biosurfactants Co., Saukville, WI, United States), and there is a number of reports regarding their antibacterial and anti-biofilm activities (Abdel-Mawgoud et al., 2010; Vatsa et al., 2010; Kim et al., 2015; Zhong et al., 2015). Di-rhamnolipids (di-Rha), as a class of rhamnolipids, are less studied but received attention as they showed therapeutic properties for wound healing and ulcer treatment (Stipcevic et al., 2006; Piljac et al., 2008). Stable rhamnolipid production has been achieved with *P. aeruginosa* and certain *Burkholderia* strains (Chrzanowski et al., 2012; Dobler et al., 2016), however, the identification and the development of other rhamnolipid producing bacteria is an important issue to avoid potential pathogenicity and to broaden the spectrum of the products.

In this study, we sought to identify secondary metabolites amongst metabolites of new bacterial isolates that would have efficient anti-biofilm activity preferably without the effect on bacterial growth, to avoid the development of resistance. As the most activity has been associated with the di-rhamnolipids, the investigation has been extended to the semi-synthetic preparation of di-rhamnolipids amide derivatives and comprehensive assessment of their anti-biofilm, antibacterial, and cytotoxic activities.

## Materials and Methods

### Materials

Chemicals and reagents including rhamnolipids mixture R90 (AGAE Technologies) and 4-(Dimethylamino)pyridine (DMAP) were purchased from Sigma–Aldrich (Munich, Germany). Reagents used for coupling reactions including *N*-(3-Dimethylaminopropyl)-*N*′-ethylcarbodiimide hydrochloride (EDCI) were purchased from ACROS Organics (Morris Plains, NJ, United States). Unless otherwise stated, all media components were purchased either from Oxoid (Cambridge, United Kingdom) or Becton Dickinson (Sparks, MD, United States).

Gentamycin sulfate and chloramphenicol were purchased from Sigma–Aldrich (Munich, Germany), tetracycline hydrochloride from USB Co. (Cleveland, OH, United States) and cycloheximide from SERVA (Heidelberg, Germany). The stock solutions of antibiotics were prepared as follows: gentamycin 30 g L^-1^ in water, tetracycline 15 g L^-1^ in ethanol, chloramphenicol 50 g L^-1^ in ethanol, and cycloheximide 70 g L^-1^ in water and were kept at -20°C.

### Isolation and Identification of Bacterium BV152.1

Bacteria were isolated from the rhizosphere of medicinal plant *Glechoma hederacea* L. (ground ivy), using previously described procedure (Djokic et al., 2011). The medium for isolation of bacteria was starch casein agar containing soluble starch (10 g L^-1^), casein (1 g L^-1^), KH_2_PO_4_ (0.5 g L^-1^), MgSO_4_ (0.5 g L^-1^), NaCl (3 g L^-1^), and bacteriological agar (15 g L^-1^), supplemented with antifungal cycloheximide (50 μg L^-1^). Plates were incubated at 30°C for 7 days.

Isolates were grown in JS broth containing soy flour (30 g L^-1^), soluble starch (20 g L^-1^), glucose (20 g L^-1^), mannitol (15 g L^-1^), and CaCO_3_ (10 g L^-1^) for 7 days, at 30°C on a rotary shaker (180 rpm) and their cultures were extracted with equal volume of ethyl acetate and screened for the inhibition of biofilm formation of *P. aeruginosa* PAO1.

Genomic DNA isolation, PCR amplification of 16S rRNA gene sequencing and sequence alignment were performed as described previously (Stankovic et al., 2012).

### Preparation and Analysis of BV152.1 Culture Extracts

Spore suspensions of BV152.1 were prepared in glycerol [20%, v/v, (Kieser et al., 2000)] (20 μl) and firstly inoculated into vegetative medium (tryptone soy broth, 8 g L^-1^, yeast extract, 4 g L^-1^, maltose, 15 g L^-1^, and CaCO_3_, 2 g L^-1^) and incubated at 30°C for 48 h, and this pre-culture was then used for the inoculation of JS broth (1%, v/v). Cultures were grown in Erlenmeyer flasks (1:5 culture to volume ratio) containing coiled stainless steel spring for better aeration and incubated at 30°C on a rotary shaker (180 rpm) for 7 days.

Culture (1 L) was extracted using the equal volume of ethyl acetate by vigorous shaking for 30 min. The organic phase was then separated, dried under vacuum and further purified by flash chromatography. Flash chromatography employed silica gel 60 (230–400 mesh) and the following solvent system: n-hexane and ethyl acetate (3:1 ratio, 100 ml), n-hexane and ethyl acetate (1:1 ratio, 100 ml), ethyl acetate and methanol (9:1 ratio, 100 ml), followed by pure methanol (100 ml). Collected fractions were analyzed by thin layer chromatography, which was carried out using alumina plates with 0.25 mm silica layer (Kieselgel 60 F_254_, Merck, Darmstadt, Germany) and the appropriate fractions were combined, dried under vacuum, and weighted. Anti-biofilm activity was determined for each fraction.

### Purification and Characterization of Di-Rhamnolipids

The fraction that showed anti-biofilm forming activity was purified further using the following solvents: pure ethyl acetate (50 mL); ethyl acetate, dichloromethane, and methanol (8:1:1 ratio, 50 mL) and ethyl acetate, dichloromethane, and methanol (4:2:1 ratio, 50 mL). The most active fraction (F3) was further analyzed by a combination of NMR and mass spectrometry. The NMR spectra were recorded on a Bruker Ascend 400 (400 MHz) spectrometer. Chemical shifts are given in parts per million (δ) downfield from tetramethylsilane as the internal standard and deuterochloroform as a solvent.

Mass spectral data were recorded using 6210 Time-of-Flight LC–MS system (Agilent Technologies, Santa Clara, CA, United States) connected to an Agilent 1200 Series HPLC instrument (Agilent Technologies, Waldbronn, Germany), with a degasser, a binary pump, an autosampler, a column compartment equipped with a Zorbax Eclipse XDB-C18 RRHT column (1.8 μm, 4.6 mm × 50 mm) and a diode-array detector, via ESI interface. The mobile phase consisted of water containing 0.2% formic acid (v/v; A) and acetonitrile (B). A gradient program was used as follows: 0–0.24 min 5% B, 0.24–10 min, 5–95% B, 10–15 min, 95% B, 15–15.5 min, 95–5% B, 15.5–20 min, 5% B. The flow rate of mobile phase was 0.5 mL/min, the column temperature was 40°C and the injection volume was 10 μL. Spectral data from all the peaks were accumulated in the range of 190–900 nm. Full scan mass spectra were measured between 100 and 1500 *m/z* in positive ion mode.

### Synthesis and Characterization of Bacterial Rhamnolipid Derivatives

To a solution of di-rhamnolipids (0.1 mmol, 1 eq.) in CH_2_Cl_2_/DMF (2.7:0.3 mL) were added EDCI (0.12 mmol, 1.2 eq.), DMAP (0.05 mmol, 0.5 eq.) and amine (benzyl amine, piperidine and morpholine; 0.3 mmol, 3 eq.). The mixture was stirred for 16 h at room temperature. The mixture was diluted with water (10 mL) and extracted with CH_2_Cl_2_ (3 × 15 mL). The combined organic extract was dried with MgSO_4_, filtered and concentrated in vacuo. The residue was purified by flash column chromatography (SiO_2_, ethyl acetate:methanol:dichloromethane in 4:2:1 ratio) that afforded products as yellow thick oils.

To obtain silylated derivative to a solution of rhamnolipids (0.1 mmol, 1 eq.) in DMF (3 mL) TBDMS-Cl (1.6 mmol, 16 eq.) AgNO_3_ (1.6 mmol, 16 eq.) and pyridine (3.2 mmol, 32 eq.) were added. The mixture was stirred overnight at ambient temperature before it was diluted with water (10 mL) and extracted with ethyl acetate (3 × 20 mL). The combined organic extract was dried with MgSO_4_, filtered and concentrated in vacuum to yield the product which was purified via silica gel flash chromatography using petroleum ether/EtOAc (7:1) as eluent to provide the title compound as a colorless oil.

Physico-chemical properties of all compounds were predicted using the Marvin Sketch 17.2.13.0 program (ChemAxon^1^). Consensus methods were used for calculating logP and hydrophilic-lipophilic balance (HLB) values.

### Test Organisms

Test organisms for the antibacterial assays were obtained from the National Collection of Type Cultures (NCTC) and the American Type Culture Collection (ATCC). *P. aeruginosa* PAO1 NCTC 10332, *S. aureus* ATCC 25923 and *S. aureus* ATCC 43300 (MRSA) and *S. marcescens* ATCC 27117 were used in this study. One clinical isolate of *P. aeruginosa* DM50 with high ability to form biofilms and resistance to metronidazole, clindamycin and amoxicillin was also included (Glisic et al., 2016). Bacterial strains were grown in Luria Bertani (LB) broth at 37°C on a rotary shaker at 180 rpm.

All rhamnolipids utilized for the bioactivity assessments in this study along with sources and descriptions are listed in Supplementary Table S1.

### Antimicrobial Susceptibility Tests for Planktonic Cells

The minimum inhibitory concentration (MIC) of rhamnolipids and their derivatives were determined according to standard broth microdilution assays recommended by the National Committee for Clinical Laboratory Standards (M07-A8).

Stock solutions of rhamnolipids and derivatives were prepared in DMSO (50 g L^-1^, w/v). The highest concentration used was 500 mg L^-1^. The inoculums were 10^5^ colony forming units (cfu) mL^-1^. The MIC value corresponds to the lowest concentration that inhibited the growth after 24 h at 37°C for *P. aeruginosa* and *S. aureus* or at 30°C for *S. marcescens*.

### Inhibition of Biofilm Formation

Biofilm quantification assays were performed in 96-well microtiter plates using a crystal violet (CV) method to stain adherent cells (Merritt et al., 2005). Overnight cultures of bacteria were diluted to 5 × 10^7^ cfu mL^-1^ in LB and 100 μL was added to the wells in the presence of test compounds or DMSO (0.1%, v/v). Biofilms formed for 24 h at 37°C for *P. aeruginosa* and *S. aureus*, or 30°C for *S. marcescens* were washed and adherent cells stained with CV 0.1% (v/v). BFIC_50_ (concentration of compound that inhibited biofilm formation by 50%) was determined for each compound.

The effect of rhamnolipids on *P. aeruginosa* biofilm formation was examined additionally by introducing 3 h adhesion phase after inoculation with 5 × 10^7^ cfu mL^-1^ cells. Following adhesion, the supernatant was removed and, after two washing steps with phosphate buffered saline (PBS), test compounds or DMSO were applied and the biofilms were quantified after 24 h using CV. Each biofilm formation assay was performed in six wells and repeated at least three times.

### Scanning Electron Microscopy (SEM)

To study the effect of di-rhamnolipids on *P. aeruginosa* PAO1 adherence to silicone surfaces and overnight bacterial culture was diluted to 5 × 10^7^ cfu mL^-1^ in LB and 2 ml was added per well in six well microtiter plate. The silicone catheter pieces (Romed, Wilnis, Holland) of 1 cm were placed in each well containing diluted bacteria and incubated in the presence of di-rhamnolipids from *Lysinibacillus* sp. BV152.1 (F3) or DMSO. After 24 h, the culture medium was removed and the catheters were washed three times in PBS in order to remove the non-adherent strains. Biofilms were then fixed with cold methanol and the samples dried before the examination.

Catheters were glued to double-sided conductive carbon tab stuck on standard vacuum-clean stub, and were coated with gold (thickness of 15–20 nm) by the sputtering process (Leica EM SCD005 sputtering machine, Leica Microsystems, Mannheim, Germany). Sputtering was performed in the vacuum chamber under pressure <0.05 mbar using sputter current of 40 mA, the working distance of 50 mm and sputter time of 100 s. Such prepared samples were examined by JEOL JSM-6610LV microscope (JEOL United States, Inc., Peabody, MA, United States). An acceleration voltage of 20 kV was used.

### Biofilm Microscopy

To study the effect of di-rhamnolipids on *P. aeruginosa* PAO1 adherence to glass or plastic surfaces biofilms were developed on cover slips and examined under the microscope. An overnight culture of *P. aeruginosa* PAO1 was diluted to 5 × 10^7^ cfu mL^-1^ in LB and 2 ml was added per well of six well microtiter plate containing glass or plastic cover slips. After 24 h, non-adherent cells were removed and biofilms were washed with 0.9% NaCl and stained with 2.5 μM SYTO9 green fluorescent dye and 2.5 μM propidium iodide (PI) red fluorescent dye of Live/Dead staining kit (LIVE/DEAD^®^ BacLight^TM^ Bacterial Viability Kit, Thermo Fisher Scientific, Waltham, MA, United States). Cells were observed under a fluorescence microscope (Olympus BX51, Applied Imaging Corp., San Jose, CA, United States) under 100,000 × magnification (glass) or 40,000 × magnification (plastic).

### Anti-adhesion Assay

Anti-adhesion assay was performed as previously described (Shetye et al., 2014) with some modifications. An overnight culture of *P. aeruginosa* PAO1 containing pBBR2-GFP (da Silva et al., 2014) was subcultured (initial OD600 = 0.02) in M9+/LB (95:5) containing gentamycin (80 mg L^-1^), tetracycline (20 mg L^-1^), and chloramphenicol (50 mg L^-1^) at 37°C in a rotary shaker (200 rpm). After reaching OD600 = 0.1, aliquots (200 μL) were transferred to the wells of black polystyrene microtiter plate with rhamnolipids, derivatives or DMSO (control). After incubation at 37°C for 2 h, bacterial cultures were discarded, and the fluorescence of adherent cells was measured after addition of fresh M9+/LB (95:5) medium using Tecan Infinite 200 Microplate reader (Tecan Group Ltd., Switzerland, lex = 500 nm, lem = 540 nm). Background signal (M9+/LB (95:5) was subtracted from all the samples. Assays were repeated three times; inhibition values are the averages of six replicate wells from one experiment.

### Biofilm Dispersion Assay

An overnight culture of *P. aeruginosa* was diluted to 5 × 10^7^ cfu mL^-1^ in LB and 100 μL was added to the wells in 96-well microtiter plates and incubated for 24 h at 37°C. After removal of the supernatant and two washing steps with PBS adherent cells were treated with different concentrations of rhamnolipids or derivatives for additional 24 h and the biofilms were quantified using CV as described above. Biofilm dispersion assays were performed in six wells and repeated three times.

### Cytotoxicity Assay

MRC5 human lung fibroblasts were obtained from the ATCC. Cells were maintained as monolayer cultures in RPMI-1640 supplemented with 100 mg L^-1^ streptomycin, 100 U mL^-1^ penicillin and 10% (v/v) FBS (all from Sigma, Munich, Germany). Cells were grown in a humidified atmosphere of 95% air and 5% CO_2_ at 37°C.

Cytotoxicity on MRC5 cells was evaluated with 3-(4,5-dimethylthiazol-2-yl)-2,5-diphenyltetrazolium bromide (MTT). The assay was carried out after 48 h of cell incubation in the media containing test compounds at different concentrations and the viability was measured as described (Mihajlovic et al., 2012). The results are presented as the percentage of the control (untreated cells) that was arbitrarily set to 100%.

### Statistical Analysis

The results were analyzed by Student’s *t*-test using SPSS version 20 software. A *P*-value lower than 0.05 was considered as statistically significant.

## Results

### Isolation and Identification of BV152.1

The rhizosphere of medicinal plant *G. hederacea* L. (ground ivy) proved a prolific source of aerobic bacteria with 32 morphologically differing isolates collected from this source (data not shown). Culture extracts from these isolates were screened for their ability to inhibit biofilm formation in *P. aeruginosa* PAO1 using CV based assay in 96-well polystyrene plates. During the preliminary screening, the crude cell extract of the isolate BV152.1 showed the most potent ability to inhibit 60% of biofilm formation when applied in the concentration of 500 μg mL^-1^ while no bactericidal activity of the extract was observed. This isolate was selected for structural characterization of its secondary metabolites as the most promising one. The isolate BV152.1 was identified as a member of the Gram-positive genus *Lysinibacillus* on the basis of 16S rRNA gene sequence analysis. Its 16S rRNA gene sequence (GenBank access No. KY933395) showed 99% similarity with type strains of *Lysinibacillus louembei* NM73 (NR_145586.1), *L. meyeri* WS 4626 (NR_117577.1), and *L. odysseyi* YH2 (KM873372.1).

### Di-Rhamnolipids As Active Components of *Lysinibacillus* sp. BV152.1 Culture Extracts

In order to identify the component with biofilm formation inhibitory activity produced by *Lysinibacillus* sp. BV152.1, 1 L culture grown for 7 days at 30°C was extracted with ethyl acetate and the total mass of 800 mg crude cell extract was obtained. The crude cell extract was subsequently fractionated, the activity of each fraction measured and the fraction designated as F3 was identified as the most active one. After further purification, the total mass of F3 was determined to be 60 mg and it showed 50% inhibition of biofilm formation when applied in a concentration of 50 μg mL^-1^ (**Table 1**).

**Table 1:** Antibacterial activity of rhamnolipids mixture (R90), di-rhamnolipids and di-rhamnolipid derivatives from *Lysinibacillus* sp. BV152.1 and *Pseudomonas aeruginosa* determined after 24 h incubation.

Rhamnolipids MIC^a^ (μg mL^-1^)	*P. aeruginosa* PAO1	*P. aeruginosa* DM50	*S. aureus* ATCC 25923	*S. aureus* MRSA	*S. marcescens* ATCC 27117
***Lysinibacillus* sp. BV152.1**					
di-Rha mixture (F3)	>500	>500	>500	>500	>500
di-Rha-Bn	>500	>500	>500	>500	>500
di-Rha-Pip	>500	>500	>500	250	>500
di-Rha-Mor	>500	>500	>500	>500	>500
di-rha-TBDMS	>500	>500	>500	>500	>500
***P. aeruginosa***					
R90	>500	>500	250	250	>500
di-Rha	>500	>500	>500	>500	>500
di-Rha-Bn	>500	>500	62.5	125	>500
di-Rha-Pip	>500	>500	62.5	62.5	>500
di-Rha-Mor	>500	>500	62.5	62.5	>500
di-Rha-TBDMS	>500	>500	>500	>500	>500


*^a^The minimum inhibitory concentration (MIC) determined as the lowest concentration of compound at which no evident growth was observed. Values are the average of two independent experiments performed in four wells.*

**FIGURE 1:**
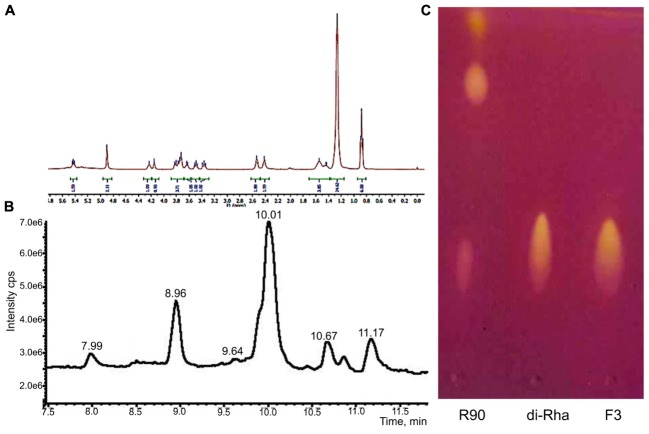
Structural characterization of di-rhamnolipids (F3) isolated from *Lysinibacillus* sp. BV152.1. **(A)** ^1^H NMR spectrum of F3, **(B)** HPLC chromatogram of F3, and **(C)** thin layer chromatography of *Pseudomonas aeruginosa* rhamnolipids (R90), purified di-rhamnolipids from R90 (di-Rha) and di-rhamnolipids fraction isolated from *Lysinibacillus* sp. BV152.1 culture (F3).

^1^H-NMR and ^13^C-NMR spectra of F3 showed the presence of fatty acid chains and carbohydrate moieties (**Figure 1A**) indicative of the characteristic spectra of bacterial di-rhamnolipids. The peak observed at δ 0.88 ppm indicated the presence of terminal methyl group and the peak at δ 1.26, characteristic for methylene group, indicated the presence of a straight-chain fatty acid. The peaks observed at δ 4.15–3.36 and peaks for two anomeric protons at δ 4.90 confirmed the presence of two carbohydrate units. The ^13^C-NMR analysis demonstrated two carbonyl peaks at δ 173.7 and 171.4, two anomeric carbon peaks at δ 102.5 and 94.5 and expected number of peaks for carbohydrate and fatty acid units. In general, the spectral characteristics obtained for F3 (provided in Supplementary material) were in good agreement with the data published in the literature for di-rhamnolipids (Sharma et al., 2007).

The exact lengths of the fatty acid chains were deduced from HPLC-MS data (**Figure 1B**). Sodiated molecular ions, [M + Na]+, in the ESI+MS data were observed at *m/z* 701 (Rha-Rha-C10-C12, 11.16 min, 9%), 699 (Rha-Rha-C10-C12:1, 10.68 min, 8%), 673 (Rha-Rha-C10-C10, 10.03 min, 63%, major), and 645 (Rha-Rha-C8-C10, 8.96 min, 17%), 527 (Rha-C10-C10, 10.86 min, 3%).

Considering the identified structural characteristics of F3, we have selected commercially available *P. aeruginosa* sp. derived rhamnolipids (R90), as the model and the control compound. R90 was determined to contain mono- vs. di-rhamnolipid form in 4:1 ratio (AGAE Technologies, **Figure 1C**) with the major component of Rha-Rha-C10-C10 in more than 90%.

### Anti-biofilm Properties of Di-Rhamnolipids

**FIGURE 2:**
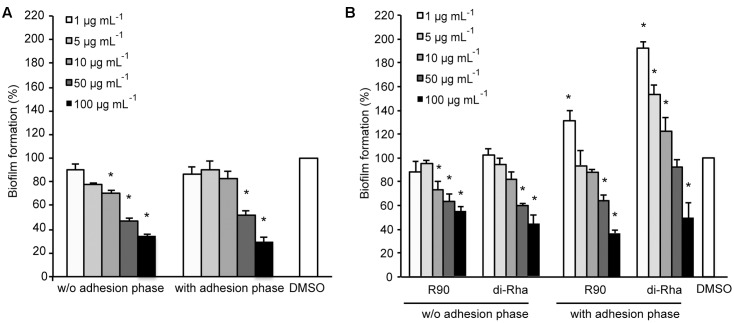
Inhibition of *P. aeruginosa* PAO1 biofilm formation with **(A)** di-rhamnolipids produced by *Lysinibacillus* sp. BV152.1 (F3) and **(B)** *P. aeruginosa* (rhamnolipids mixture and purified di-Rha). ^∗^*P* < 0.05.

Di-rhamnolipids from *Lysinibacillus* sp. BV152.1 (F3) containing mainly di-rhamnolipids with Rha-Rha-C10-C10 63% showed dose-dependent anti-biofilm formation activity in *P. aeruginosa* PAO1 with 50 μg mL^-1^ determined as BFIC_50_ (**Figure 2A**). In order to assess whether di-rhamnolipids from *Lysinibacillus* sp. BV152.1 affected biofilm growth, *P. aeruginosa* PAO1 cells were left to attach to the surface of microtiter wells for 2 h prior to incubation with F3 for 24 h. Inhibition of biofilm formation was similar in the presence or absence of the cell adhesion phase showing that di-rhamnolipids affected both cell attachment and biofilm growth (**Figure 2A**).

Anti-biofilm properties of di-rhamnolipids from *Lysinibacillus* sp. BV152 were compared to that of commercially available rhamnolipids obtained by fermentation of *P. aeruginosa* sp. (R90) (**Figure 2B**). Di-rhamnolipids (di-Rha) from this sample were also purified and used for the comparison. Rhamnolipids mixture (R90) and purified di-Rha from *P. aeruginosa* showed slightly lower activity against *P. aeruginosa* PAO1 biofilm formation (BFIC_50_ = 75 μg mL^-1^) comparing to F3. The effects of R90 and di-Rha on biofilm formation were comparable when the treatments were applied without cell adhesion phase. However, when bacteria were left to attach to the surface prior to treatments, di-Rha stimulated biofilm formation when added in concentrations up to 50 μg mL^-1^. Di-rhamnolipids from both sources showed an equal potency in inhibition of biofilm formation at concentrations above 50 μg mL^-1^.

**FIGURE 3:**
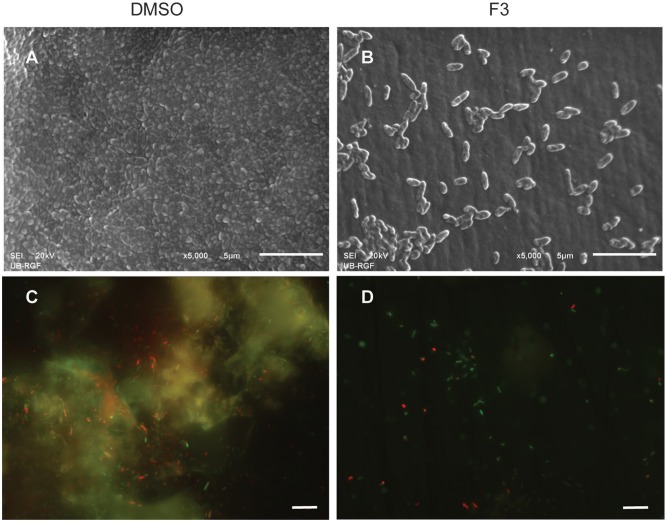
Inhibition of cell attachment and biofilm formation with di-rhamnolipids from *Lysinibacillus* sp. BV152.1. Biofilms *P. aeruginosa* PAO1 were formed for 24 h on silicone catheter **(A,B)** or glass **(C,D)** in the presence of DMSO (0.1%) or F3 (50 μg mL^-1^). Biofilms were analyzed by scanning electron microscopy (SEM; **A,B**) or fluorescent microscopy **(C,D)**. In **(C,D)** bacteria labeled with Syto9 appeared green and bacteria stained with propidium iodide (PI) are red, scale bars represent 10 μm.

Di-rhamnolipids from *Lysinibacillus* sp. BV152.1 efficiently inhibited bacterial adhesion to the polystyrene surface of microtiter plates and also to the surface of silicone catheters or glass coverslips as visualized by SEM and fluorescent microscopy, respectively (**Figure 3**).

Production of rhamnolipids in *P. aeruginosa* enables its swarming motility (Caiazza et al., 2005). Therefore, the effect of di-rhamnolipids on *P. aeruginosa* PAO1 swarming motility was analyzed next and the results showed that both F3 and R90 stimulated swarming in dose-dependent manner (Supplementary Figure S1). The observed effect was more prominent in the presence of di-rhamnolipids from *Lysinibacillus* sp. BV152.1.

### Derivatization of Di-Rhamnolipids and Their Antibacterial and Cytotoxic Properties

**FIGURE 4:**
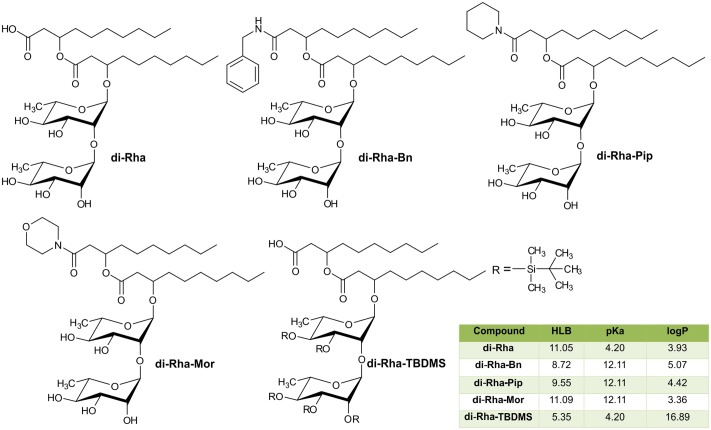
Chemical structures of di-Rha (C10-C10) and amide derivatives synthesized in this study with calculated parameters hydrophilic-lipophilic balance (HLB), acid dissociation constant (pKa), partition coefficient (logP).

The semi-synthetic approach of generating amide derivatives from two different sources of di-Rha was straightforward, with products obtained in high purity and yields from 25 to 55% (**Figure 4**). Products were characterized using NMR and LC-MS (Supplementary Figures S2–S5). In addition, silylated derivative (di-Rha-TBDMS) was also generated for the control purposes. Physico-chemical parameters for all compounds were calculated revealing the HLB value ranging from 8.72 to 11.09 for di-Rha and amide derivatives, while this value for di-Rha-TBDMS was 5.35 (**Figure 4**). It is known that compounds with HLB 7–11 are considered wetting agents and water in oil emulsifiers, while the ones having HLB between 4 and 6 as water in oil emulsifiers (Pasquali et al., 2008; Muller et al., 2012). pKa was three times higher for amide derivatives in comparison to di-Rha and di-Rha-TBDMS indicating that they were weaker acids. logP was between 3.36 and 5.07 for di-Rha and amide derivatives, while this value for di-Rha-TBDMS was 3–5 times higher, suggesting higher hydrophilicity of amide derivatives (**Figure 4**). Spectral data for the new compounds are provided in Supplementary material.

Antibacterial activity of all derivatives obtained from both rhamnolipids sources against *P. aeruginosa* PAO1, *P. aeruginosa* DM50, *S. aureus* ATCC 25923, *S. aureus* MRSA and *S. marcescens* was addressed. Neither F3 nor di-Rha derivatives obtained from *Lysinibacillus* sp. BV152.1 exhibited antibacterial activity at concentrations up to 500 μg mL^-1^ against any of the bacterial species tested (**Table 1**). None of the derivatives synthetized from *P. aeruginosa* di-Rha affected *P. aeruginosa* and *S. marcescens* growth. However, *P. aeruginosa* derivatives di-Rha-Pip and di-Rha-Mor exhibited bactericidal activity against *S. aureus* ATCC 25923 strain and *S. aureus* MRSA with MIC concentrations 62.5 μg mL^-1^. Derivative Rha-Bn from *P. aeruginosa* demonstrated the same antibacterial activity against *S. aureus* MRSA.

**FIGURE 5:**
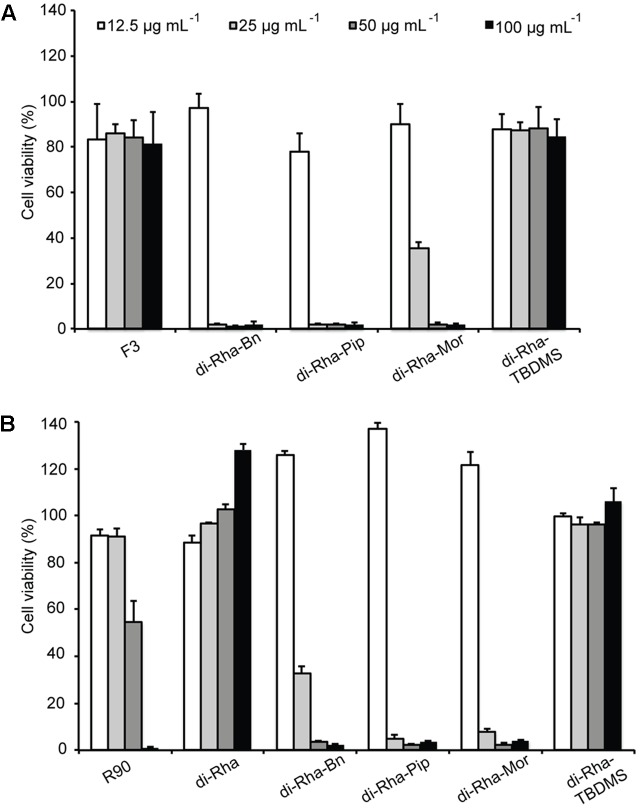
Cytotoxic effects of rhamnolipids mixtures, di-rhamnolipids and their derivatives from *Lysinibacillus* sp. BV152.1 **(A)** and *P. aeruginosa* **(B)** on human fibroblasts (MRC5) measured by MTT method, following 48 h exposure. Values are representative of two independent experiments ± SD.

Cytotoxicity of rhamnolipids mixture (R90), di-Rha mixture (F3), purified di-Rha and their derivatives was analyzed (**Figure 5**). Viability of human lung fibroblasts (MRC5) was not affected in the presence of F3, di-Rha and di-Rha-TBDMS when applied in concentrations up to 100 μg mL^-1^. Rhamnolipids mixture R90 showed cytotoxic effect with IC_50_ value 50 μg mL^-1^. Derivatization of di-Rha substantially increased their cytotoxicity reaching almost 100% cells killing at concentrations of 25 μg mL^-1^. These concentrations were 2.5-fold lower than their MIC concentration values against *S. aureus* strains (**Table 1**) and potentially are limiting factor in their further development as antibiotics.

### Anti-biofilm Activity of Di-Rhamnolipids and Their Amide Derivatives

**FIGURE 6:**
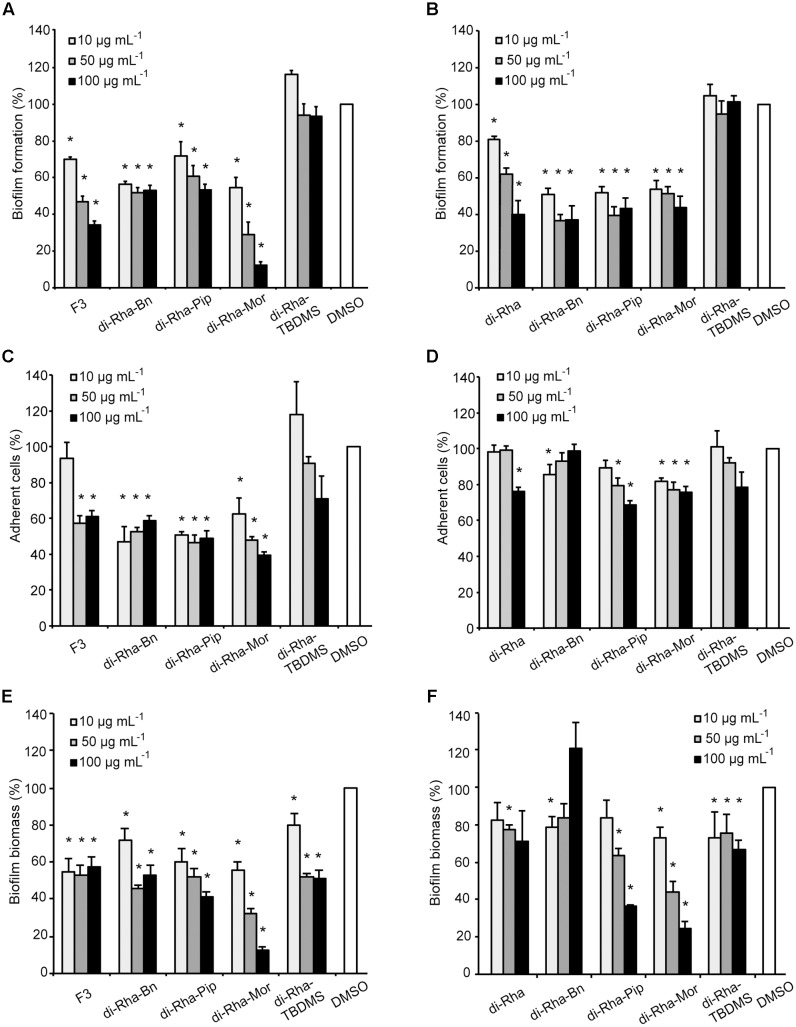
*Pseudomonas aeruginosa* PAO1 biofilm formation **(A,B)**, cell adhesion **(C,D)**, or biofilm disruption (**E,F**; %) in the presence of di-rhamnolipids isolated from *Lysinibacillus* sp. BV152.1 **(A,C,E)** or *P. aeruginosa* **(B,D,F)** and their derivatives. Values are presented as mean ± SD. ^∗^*P* < 0.05.

**FIGURE 7:**
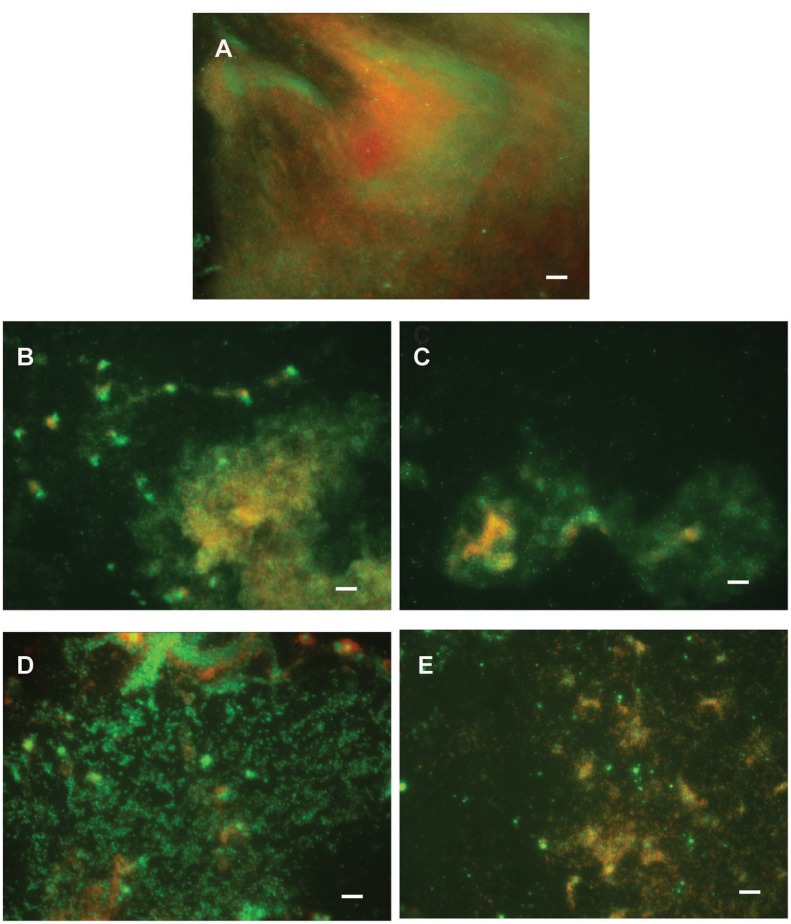
*Pseudomonas aeruginosa* PAO1 biofilm formation on plastic surfaces in the presence of 0.1% DMSO **(A)**, F3 **(B)**, di-Rha-Mor derivative from *Lysinibacillus* sp. BV152.1 **(C)**, or di-Rha **(D)**, and di-Rha-Mor derivative from *P. aeruginosa* **(E)** at 50 μg mL^-1^. Biofilms were stained with Syto9 (green) and PI (red), scale bars represent 10 μm.

Anti-biofilm properties of di-rhamnolipids and their derivatives were examined by addressing their influence on biofilm formation, cell adhesion and disruption of pre-formed biofilms. Biofilm formation in *P. aeruginosa* PAO1 was quantified in the presence or absence of di-rhamnolipids and their derivatives. Derivative di-Rha-Mor from *Lysinibacillus* sp. BV152.1 showed increased anti-biofilm formation activity compared to di-rhamnolipids mixture F3 (BFIC_50_ = 12.5 μg mL^-1^) inhibiting 90% biofilm formation at 100 μg mL^-1^ (**Figures 6A**, **7**). Anti-biofilm formation activity of di-Rha-Bn and di-Rha-Pip was twofold lower than that of F3 (BFIC_50_ = 100 μg mL^-1^). Amide derivatization of di-Rha from *P. aeruginosa* significantly improved activities of all derivatives with BFIC_50_ = 10 μg mL^-1^ (**Figures 6B**, **7**). However, maximum activity was reached already at 50 μg mL^-1^ with biofilm formation inhibition up to 60%. Derivatives di-Rha-TBDMS from both sources, showed no anti-biofilm formation activity.

To measure anti-adhesion activity of di-rhamnolipids and their amide derivatives, GFP fluorescence of *P. aeruginosa* PAO1-GFP was used to quantify bacterial adhesion 2 h after inoculation. Relative to the amount of adhered bacteria without treatment (containing 0.1% DMSO), derivatives di-Rha-Bn and di-Rha-Pip from *Lysinibacillus* sp. BV152.1 inhibited PAO1-GFP adhesion by 50% at concentrations 10 μg mL^-1^, while di-Rha-Mor showed 50% adhesion inhibition at 50 μg mL^-1^ (**Figure 6C**). Again, derivatives from *P. aeruginosa* exhibited less prominent anti-adhesive activities inhibiting *P. aeruginosa* PAO1-GFP adhesion up to 35% at concentrations 100 μg mL^-1^ (**Figure 6D**). Derivative di-Rha-TBDMS showed no significant anti-adhesion activity. Importantly, di-Rha and their derivatives did not affect GFP expression nor quenched its fluorescence (Supplementary Figure S10).

**FIGURE 8:**
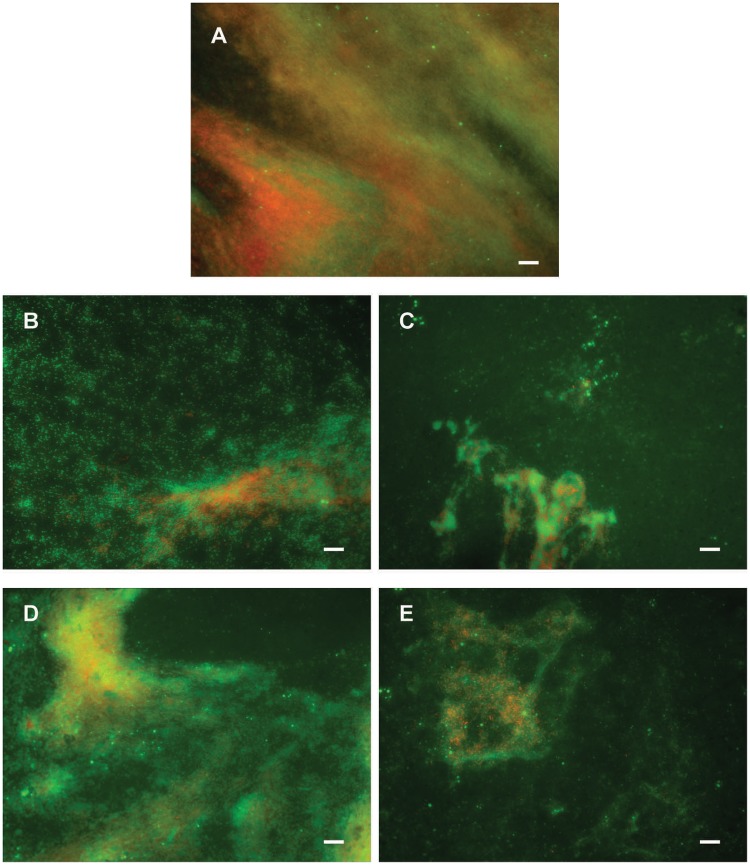
Dispersion of *P. aeruginosa* PAO1 biofilms pre-formed on plastic surfaces **(A)** with 50 μg mL^-1^ F3 **(B)**, di-Rha-Mor derivative from *Lysinibacillus* sp. BV152.1 **(C)**, di-Rha **(D)**, or di-Rha-Mor derivative from *P. aeruginosa* **(E)**. Biofilms were stained with Syto9 (green) and PI (red), scale bars represent 10 μm.

Potential to disperse formed biofilm is often more relevant to medical applications, and more challenging than inhibition of biofilm formation. Therefore, the ability of di-rhamnolipids and their amide derivatives to disperse 24 h old *P. aeruginosa* biofilms was examined next (**Figures 6E,F**, **8**). Di-rhamnolipids from *Lysinibacillus* sp. BV152.1 (F3) were more effective in biofilm disruption with BDIC_50_ 10 μg mL^-1^ (BDIC_50_ is the concentration of compound that caused 50% biofilm disruption) comparing to di-Rha from *P. aeruginosa* which disrupted biofilms up to 20% at concentrations 100 μg mL^-1^. The strongest biofilm dispersion activity was measured for di-Rha-Mor derivatives from both sources, with BDIC_50_ values 12.5 and 50 μg mL^-1^ from *Lysinibacillus* sp. BV152.1 and *P. aeruginosa*, respectively. Both di-Rha-Mor derivatives were able to disrupt more than 80% of pre-formed biofilms when applied at 100 μg mL^-1^.

Taken together, these results demonstrated that amide derivatization improved di-rhamnolipids anti-biofilm properties, with di-Rha-Mor derivative being the most active compound. The results were confirmed by comparing anti-biofilm formation activities of di-rhamnolipids and their amide derivatives against different bacterial species (**Tables 2**, **3**). The improvement of rhamnolipids anti-biofilm formation activity with amide derivatization was more prominent with di-rhamnolipids from *Lysinibacillus* sp. BV152.1. Anti-biofilm formation activity of these derivatives was species specific with the highest activity observed for di-Rha-Bn and di-Rha-Pip against *S. aureus* ATCC 25923 (>90% inhibition). All three derivatives were active against *S. marcescens* biofilm formation (>80%), while the highest activity against *P. aeruginosa* biofilms was observed for di-Rha-Mor inhibiting 80% biofilm formation. The most active *P. aeruginosa* di-Rha derivative was also di-Rha-Mor, with improved anti-biofilm formation activity compared to di-Rha against *S. aureus* and *S. marcescens* (70% and 85% inhibition at 50 μg mL^-1^, respectively). The strongest inhibition of *P. aeruginosa* PAO1 biofilm formation was achieved with *P. aeruginosa* di-Rha-Bn and di-Rha-Pip treatments, while none of the derivatives affected biofilm formation in clinical isolate *P. aeruginosa* DM50 (**Table 2**). Derivatization of di-rhamnolipids from both sources also improved their biofilm dispersion activity against *P. aeruginosa* DM50, *S. aureus* ATCC 25923 and *S. aureus* MRSA (**Table 3**). Dispersion of *S. marcescens* biofilms was efficient only with di-Rha-Bn and di-Rha-Mor from *Lysinibacillus* sp. BV152.1 (63% and 58% biofilm dispersion at 50 μg mL^-1^, respectively). Di-Rha from both sources and the other derivatives showed no biofilm dispersion activity against this bacterium, most likely be due to slightly different carbon chain length between di-rhamnolipid sources.

**Table 2:** Biofilm formation (%) in the presence of rhamnolipids mixture (R90), di-rhamnolipids, and di-rhamnolipid derivatives from *Lysinibacillus* sp. BV152.1 and *P. aeruginosa*.

Rhamnolipids 50 μg mL^-1^	*P. aeruginosa* PAO1	*P. aeruginosa* DM50	*S. aureus* ATCC 25923	*S. aureus* MRSA	*S. marcescens* ATCC 27117
***Lysinibacillus* sp. BV152.1**					
di-Rha mixture (F3)	50 ± 5	45 ± 3	78 ± 12	80 ± 3	38 ± 13
Rha-Bn	60 ± 2	42 ± 2	4 0.5	66 ± 7	20 ± 4
Rha-Pip	50 ± 2	48 ± 4	4 0.5	67 ± 5	20 ± 4
Rha-Mor	20 ± 3	35 ± 8	11 ± 2	28 ± 2	12 ± 1
Rha-TBDMS	115 ± 10	100 ± 8	88 ± 12	106 ± 4	85 ± 1
***P. aeruginosa***					
R90	54 ± 5	76 ± 8	43 ± 6	42 ± 3	15 ± 2
di-Rha	50 ± 3	109 ± 10	37 ± 4	44 ± 11	20 ± 5
di-Rha-Bn	36 ± 3	168 ± 3	39 ± 2	48 ± 10	50 ± 7
di-Rha-Pip	39 ± 4	94 ± 13	31 ± 2	34 ± 3	20 ± 3
di-Rha-Mor	50 ± 4	76 ± 7	27 ± 2	30 ± 3	14 ± 1
di-Rha-TBDMS	95 ± 7	73 ± 6	88 ± 10	69 ± 11	117 ± 4


*Values are presented as mean ± SD.*

**Table 3:** Biofilm biomass (%) remained after biofilm dispersion with di-rhamnolipids and di-rhamnolipid derivatives from *Lysinibacillus* sp. BV152.1 and *P. aeruginosa*.

Rhamnolipids 50 μg mL^-1^	*P. aeruginosa* PAO1	*P. aeruginosa* DM50	*S. aureus* ATCC 25923	*S. aureus* MRSA	*S. marcescens* ATCC 27117
***Lysinibacillus* sp. BV152.1**					
di-Rha mixture (F3)	53 ± 5	108 ± 7	115 ± 13	88 ± 7	243 ± 3
Rha-Bn	46 ± 2	74 ± 4	64 ± 5	62 ± 11	37 ± 2
Rha-Pip	52 ± 5	67 ± 4	87 ± 5	86 ± 10	180 ± 7
Rha-Mor	32 ± 2	75 ± 8	68 ± 10	63 ± 12	42 ± 4
Rha-TBDMS	80 ± 6	65 ± 1	110 ± 22	101 ± 14	99 ± 8
***P. aeruginosa***					
di-Rha	77 ± 3	135 ± 10	80 ± 9	82 ± 7	193 ± 8
di-Rha-Bn	84 ± 8	70 ± 6	62 ± 5	55 ± 10	239 ± 15
di-Rha-Pip	63 ± 4	59 ± 5	80 ± 7	54 ± 2	175 ± 18
di-Rha-Mor	44 ± 6	67 ± 8	44 ± 4	70 ± 6	160 ± 8
di-Rha-TBDMS	76 ± 10	78 ± 10	82 ± 3	120 ± 10	155 ± 3


*Values are presented as mean ± SD.*

## Discussion

Rhamnolipids are amphiphilic glycolipids biosynthesized by bacteria that, due to their low toxicity and biodegradability, are potential replacements for synthetic surfactants. For *P. aeruginosa*, secretion of rhamnolipids is critical for biofilm formation, dispersion of bacteria from the mature biofilms and for their swarming motility (Davey et al., 2003; Nickzad and Deziel, 2014; Wittgens et al., 2016). Rhamnolipids are mainly produced by species of *P. aeruginosa*, ubiquitous opportunistic pathogen, which makes the isolation of novel safer natural producers an important task. In the present study, during an effort of identification of novel bacterial secondary metabolites with anti-biofilm activity, a new rhamnolipid producing bacterial strain has been isolated from the plant rhizosphere and identified as *Lysinibacillus* sp. BV152.1. Members of this Gram-positive genus, with the ability to form endospores under harsh environmental conditions, have been isolated from various environments, however, they are often found in soil and in association with plants (Ouoba et al., 2015; Fredsgaard et al., 2017; Govindasamy et al., 2017; Yan et al., 2017). *L. fusiformis* S9, isolated from the river bank soil sample in India, has been reported to produce glycolipids with surfactant properties, but the identity of the sugar moiety was not confirmed and in contrast to BV152.1 isolate, the unsaturated alkanoic acid was predominant lipid chain (Pradhan et al., 2014). Therefore this is the first report that undoubtedly confirmed the production of rhamnolipids by a strain belonging to genus *Lysinibacillus*. Some bacteria are known to produce only mono-rhamnolipids, while some produce mono- and di-rhamnolipids in various ratios, depending on the culture conditions. Here, anti-biofilm activity guided fractionation and purification of the bacterial culture extract lead toward the identification of pure di-Rha fraction (**Figure 1**).

It is well documented that rhizosphere microorganisms show wide ability to produce secondary metabolites with the pronounced antagonistic activity toward plant pathogens (Compant et al., 2005). Other bacterial isolates from the plant rhizosphere producing rhamnolipids have been isolated, however, they mostly belonged to *Pseudomonas* genus (Sharma et al., 2007). Rhamnolipid production level of *Pseudomonas* sp. GRP3 was comparable to production of di-Rha in *Lysinibacillus* sp. BV152.1 (0.041 vs. 0.06 g L^-1^), however, *Pseudomonas* sp. GRP3 mainly produced mono-Rha. The di-rhamnolipid fraction of this strain had similar composition to *Lysinibacillus* sp. BV152.1, with Rha-Rha-C10-C10 being the most prominent (87%), followed by Rha-Rha-C8-C10 and Rha-Rha-C10-C12 (Sharma et al., 2007). In general, the principal rhamnolipids considered to be produced by *P. aeruginosa* are Rha-C10-C10 and Rha-Rha-C10-C10 (Maier and Soberón-Chávez, 2000; Muller et al., 2010). Rhamnolipids can be commercially produced by *P. aeruginosa* at the level of 100 g L^-1^, upon extensive optimizations of fermentation conditions (Maier and Soberón-Chávez, 2000; Muller et al., 2012; Dobler et al., 2016). The fatty acid chain may vary from 8 to 14 carbon molecules (Abdel-Mawgoud et al., 2010). However, it was shown that biotechnologically obtained β-hydroxy alkanoic, of this carbon chain range, acids have moderate antimicrobial activity, but can be a good platform for synthesis of non-toxic molecules with improved antimicrobial properties (Radivojevic et al., 2016).

Biosurfactants, including rhamnolipids, affect the initial attachment on various surfaces, thus help prevention of the biofilm formation (Neu, 1996; Vatsa et al., 2010; Sodagari et al., 2013; Zhong et al., 2015). It was demonstrated that rhamnolipids (mixture of Rha-C10-C10 and di-Rha-C10-C10) could prevent the attachment of *P. aeruginosa*, *P. putida*, and *Escherichia coli*, as well as *S. epidermidis* and *Bacillus subtilis* on glass and octadecyltrichlorosilane modified hydrophobic glass in concentration range from 10 to 200 μg mL^-1^ to various extent (Sodagari et al., 2013). Inhibition of microbial growth, as well as a change of cell surface hydrophobicity was examined as a potential mechanism for this activity, but the responsible mechanism of the observed effect remained unknown. In the case of di-Rha utilized in this study, the antimicrobial effect has not been observed for concentrations up to 500 μg mL^-1^, while from the comparison of anti-biofilm activity with and without cell adhesion phase, it can be concluded that at concentrations of 50 μg mL^-1^ and above di-Rha reduced cell adhesion, while at lower concentrations affected biofilm maturation. Similarly, glycolipids from *L. fusiformis* S9 were found to inhibit bacterial attachment and caused the complete inhibition of *E. coli* and *Streptococcus mutans* biofilm formation at 40 μg mL^-1^ without affecting their growth (Pradhan et al., 2014). Di-Rha from both sources efficiently inhibited biofilm formation on different microorganisms including two antibiotic-resistant *S. aureus* MRSA and clinical isolate *P. aeruginosa* DM50 strains and were efficient on two types of surfaces, silicone catheter and glass. Both of these findings have great value for the possible future applications in biomedicine.

Previously limited access to relatively pure rhamnolipid materials at the gram scale has hindered extensive characterization of rhamnolipid structure-activity relation. Here, an efficient semi-synthetic methodology has been applied to the mixture and pure di-Rha, yielding three amide derivatives that were prepared and subsequently characterized for the first time. Chemical derivatization of the natural products is not a new concept, but has not been previously applied on di-Rha substrates. Indeed, based on our results, it should be further explored as a platform for obtaining new diverse rhamnolipids with improved properties. It is worth mentioning that similar semi-synthetic approach was previously applied using natural sophorolipid mixture (another class of biological glycolipids) as a substrate in reaction with the sodium alkoxides to form the corresponding sophorolipid alkyl (methyl, ethyl, propyl, and butyl) esters derivatives (Zhang et al., 2004).

A series of 14 synthetic rhamnolipids, inspired by the natural Rha-C14-C14, naturally produced by *Burkholderia plantarii*, has been achieved using hydrophobically assisted switching phase synthesis and their physico-chemical properties, as well as bioactivity in terms of cytokine induction, were examined (Howe et al., 2006). Derivatives differed in acylation pattern, number of monosaccharide residues, and the charge resulting in a large variety of activities, from the complete lost to antagonistic activity. Indeed, generating three aromatic amide derivatives from two different sources of di-Rha, we have observed a general increase in antimicrobial, anti-biofilm formation, as well as biofilm disruption and antiproliferative activities. Carboxylic group of the bioactive compounds is often modified into various amide derivatives in order to mask free carboxylic group, commonly cause an increase in lipophilicity and tailor their biological activity (Chhikara et al., 2011; Guan et al., 2014). This was especially true for the di-Rha-Mor, that was determined to be the most potent derivative, and the only one that showed the antibacterial effect against both strains of *S. aureus* with MIC concentration values of 62.5 μg mL^-1^ (**Table 1**). Similarly, the introduction of morpholine moiety resulted in the increase of activity of 4-oxo-4*H*-pyrido[1,2-*a*]pyrimidine in its ability to potentiate the activity of Levofloxacin and Aztreonam against *P. aeruginosa* (Yoshida et al., 2006). Generally, morpholine derivatives are gaining considerable importance due to diverse biological activities including antibacterial and antiproliferative (Jakubowska et al., 2008; Hirokawa et al., 2009; Seelolla et al., 2014). Relatively small differences in the structure of the three amide derivatives generated in this study caused different biological effect. On the other side, the difference in the activities between rhamnolipid compounds from two sources was small and could be attributed to the fact that derivatives from the *Lysinibacillus* sp. BV152.1 contained the mixture of congeners of various carbon chain fatty acids, while the one from *P. aeruginosa* was pure C10-C10. Interestingly, derivative di-Rha-TBDS bearing bulky protective groups on the rhamnoses lost all the biological activities, suggesting that sugar moieties play a crucial role in the activity of these molecules. Indeed, this derivative had the lowest HLB and the highest logP amongst studied molecules suggesting the loss of amphiphilic nature of rhamnolipid molecules.

Rhamnolipids are usually considered as non-toxic, however, several reports confirmed that in concentrations of 100 and 150 μg mL^-1^ they reveal significant toxicity and their antitumor activity has been focused on (Christova et al., 2013; Jiang et al., 2014). Rhamnolipids were reported to show considerable cytotoxicity on HeLa cells at a low concentration of 5 mg L^-1^ (Lotfabad et al., 2010). However, we found that di-Rha were not toxic to normal human fibroblasts up to 100 μg mL^-1^, while the antiproliferative properties increased when cells were treated with amide derivatives. This is in line with the recent study confirming that rhamnolipids, like chemical surfactants, exhibited cytotoxicity by reducing the surface tension of culture medium rather than by changing its specific molecular structure, which had no selection on tumor cells and that natural rhamnolipids are not promising antitumor agents (Jiang et al., 2014), however, simple chemical alterations may increase their cytotoxicity. We also confirm findings that natural rhamnolipids have no direct antibacterial activity in concentrations of up to 500 μg mL^-1^ against *P. aeruginosa*, *S. aureus*, and *S. marcescens*, while others have reported MIC values in a range 4–32 mg L^-1^ against bacteria *Enterobacter aerogenes*, *Proteus mirabilis*, *P. aeruginosa*, *Salmonella* Typhimurium, *S. aureus*, *B. cereus*, *B. subtilis*, *Streptococcus faecalis* and numerous fungal strains (Benincasa et al., 2004; Vatsa et al., 2010). Nevertheless, the most prominent activity of rhamnolipids is within their anti-biofilm properties that have been confirmed in numerous studies, involving wide range of rhamnolipid producers and pathogenic biofilm forming strains (Irie et al., 2005; Dusane et al., 2011, 2012; De Rienzo and Martin, 2016; Díaz De Rienzo et al., 2016). Similar to activities observed in this work, purified di-rhamnolipids from *P. aeruginosa* ATCC 9027 completely disrupted *P. aeruginosa* PAO1 pre-formed biofilms at concentrations 150 μg mL^-1^ (Davey et al., 2003). Less efficient were *P. aeruginosa* rhamnolipids against *B. pumilus* biofilms with BDIC_50_ = 580 μg mL^-1^ and complete biofilm inhibition at 50 mg mL^-1^ (Dusane et al., 2010). Generally lower in comparison to activities reported in this study but still potent biofilm disruption activities against important oral pathogens [*S. oralis* (BDIC_70_ = 750 μg mL^-1^), *Actinomyces naeslundii*, *Neisseria mucosa*, and *S. sanguinis* (BDIC_90_ = 190 μg mL^-1^)] and biofouling strains *S. capitis* and *B. licheniformis* (BDIC_50_ = 40 μg mL^-1^) were reported for rhamnolipids mixture purified from *B. thailandensis* containing mainly long chain Rha-Rha-C14-C14 di-rhamnolipids (Chebbi et al., 2017; Elshikh et al., 2017). Biofilm-related infections have been encountered in chronic diseases such as cystic fibrosis, otitis media, ventilator-associated pneumonia, and periodontitis, or in chronic wounds that have an impaired blood supply. Biofilms are often implicated in low sensitivity or resistance to antimicrobials. Thus, considering their broad-spectrum activity against bacteria and low cytotoxicity to human cells, di-Rha could be used to improve the effectiveness of antibiotics through biofilm inhibition, particularly for the treatment of chronic wound infections or in prophylactics of periodontal diseases. Besides, biofilms can be formed on the surface of medical devices (Bryers, 2008). Di-Rha and their derivatives could be used to functionalize the materials for medical usage in order to inhibit bacterial adhesion and formation of the biofilms on their surfaces, thus minimizing the spreading of the bacteria to the patients.

Recently, a series of synthetic rhamnolipid analogs including disaccharide maltose or cellobiose tethered with different aliphatic chains were prepared and their effect on the biofilm formation in *P. aeruginosa* rhamnolipid non-producing strain has been assessed (Zheng et al., 2017). As in our study, it has been shown that small structural details of these molecules are important for the bioactivities. Synthetic structural analogs of rhamnolipids promoted biofilm formation by non-rhamnolipid producing mutant at low concentrations but inhibited the biofilm formation at high concentrations such as 170 and 220 μg mL^-1^ (Zheng et al., 2017). Promotion of biofilm formation has not been observed during our study, while efficient inhibition of biofilm formation was observed at 10 and 50 μg mL^-1^.

## Conclusion

Anti-biofilm activity guided screening of culture extracts lead to the identification of rhizosphere isolate *Lysinibacillus* sp. BV152.1 producing a mixture of di-rhamnolipids. The di-rhamnolipid fraction was found to be a potent anti-biofilm agent for the pathogenic *P. aeruginosa* PAO1 and DM50, *S. aureus* (ATCC 25923 and MRSA) and *S. marcescens* ATCC 27117 strains, while having no effect on the bacterial growth and showing low *in vitro* cytotoxicity against human fibroblasts. Isolated di-Rha from *Lysinibacillus* sp. BV152.1 and from the commercially available rhamnolipid mixture from *P. aeruginosa* were used as substrates for generation of semi-synthetic amide derivatives, for the first time. Introducing amide functional group resulted in a general increase of biological activities. Thus, the semi-synthetic approach could be further explored for obtaining diverse rhamnolipids with improved activities.

## Author Contributions

All authors listed have made a substantial, direct and intellectual contribution to the work, and approved it for publication. LS and JN-R designed overall research and wrote the paper.

## Conflict of Interest Statement

The authors declare that the research was conducted in the absence of any commercial or financial relationships that could be construed as a potential conflict of interest.

## Footnotes

1 http://www.chemaxon.com
