# Supplementary material for: Anti-biofilm Properties of Bacterial Di-Rhamnolipids and Their Semi-Synthetic Amide Derivatives
Source: Front Microbiol. 2017 Dec 8;8:2454. doi: 10.3389/fmicb.2017.02454 (PMC5727045; doi:10.3389/fmicb.2017.02454)
Supplement: Supplementary file 1 [file Data_Sheet_1.PDF]

Supplementary Material

Anti-biofilm properties of bacterial di-rhamnolipids and their semi-synthetic amide derivatives

Ivana Aleksic[1], Milos Petkovic[2], Milos Jovanovic[2], Dusan Milivojevic[1], Branka Vasiljevic[1], Jasmina Nikodinovic-Runic[1*] and Lidija Senerovic[1*]

*** Correspondence:**
Lidija Senerovic
seneroviclidija@imgge.bg.ac.rs

Jasmina Nikodinovic-Runic
jasmina.nikodinovic@imgge.bg.ac.rs

1 Supplementary methods

1.1 Swarming assay

Swarm agar plates were made with M8 medium (Na_2HPO_4 (50 mM), KH_2PO_4 (25 mM), NaCl (4 mM)), supplemented with glucose (0.2%, w/v), casamino acid (0.5%, w/v), $MgSO_4$ (1 mM), and solidified with Bacto agar (0.5%, w/v) in the presence of different amounts of rhamnolipids, and inoculated with bacterial culture (3 µL, OD600=0.4–0.6) (Ha et al., 2014). Swarm agar plates were incubated at 37 °C for 24 h. The experiment was repeated two times.

1.2 Effects of compounds on GFP expression

An overnight culture of *P. aeruginosa* PAO1 containing pBBR2-GFP (da Silva et al., 2014) was subcultured (initial OD600=0.1) in LB containing gentamycin (80 mg L^{-1}), tetracycline (20 mg L^{-1}) and chloramphenicol (50 mg L^{-1}) with di-rhamnolipids from *P. aeruginosa*, their derivatives or DMSO (control) at 37 °C for 24 h in a rotary shaker (100 rpm). The culture from each well was then transferred to a flat-bottom, 96-well plate with black wall and the fluorescence and OD_{600} absorbance in each well were measured using Tecan Infinite200 multiplate-reader (Tecan Group Ltd., Switzerland, lex=500 nm, lem=540 nm). Background signals from LB broth were eliminated from all samples.

1.3 Effects of compounds on GFP fluorescence

An overnight culture of *P. aeruginosa* PAO1 containing pBBR2-GFP was washed with PBS buffer and concentrated three times in the same buffer before sonification with 4 cycles of 15 sec pulse and 15 sec rest (MSE Soniprep 150, London, UK). After centrifugation to remove cellular debris, the cell-free extract was incubated with di-rhamnolipids from *P. aeruginosa*, their derivatives or DMSO for 2h. Each sample was then transferred to a flat-bottom, 96-well plate with black wall and the fluorescence in each well was measured as described above.

2 Supplementary Figures and Tables

Table S1. Rhamnolipids and their derivatives utilized in this study.

Rhamnolipid	Description	Source/Comment
F3	Di-rhamnolipids mixture	*Lysinibacillus* sp. BV152.1 (environmental isolate described in this study)
Rha-Bn	Benzyl amide of F3	*Lysinibacillus* sp. BV152.1
Rha-Pip	Piperidine amide of F3	*Lysinibacillus* sp. BV152.1
Rha-Mor	Morpholine amide of F3	*Lysinibacillus* sp. BV152.1
Rha-TBDMS	*t*-butyldimethylsilyl protected F3	*Lysinibacillus* sp. BV152.1
R90	Rhamnolipids mixture	*Pseudomonas aeruginosa* sp. (AGAE Technologies)
di-Rha	Purified di-rhamnolipids from R90	*Pseudomonas aeruginosa* sp. (AGAE Technologies)
Rha-Bn	Benzyl amide of di-Rha purified from R90	*Pseudomonas aeruginosa* sp. (AGAE Technologies)
Rha-Pip	Piperidine amide of di-Rha purified from R90	*Pseudomonas aeruginosa* sp. (AGAE Technologies)
Rha-Mor	Morpholine amide of di-Rha purified from R90	*Pseudomonas aeruginosa* sp. (AGAE Technologies)
Rha-TBDMS	*t*-butyldimethylsilyl protected di-rhamnolipids purified from R90	*Pseudomonas aeruginosa* sp. (AGAE Technologies)

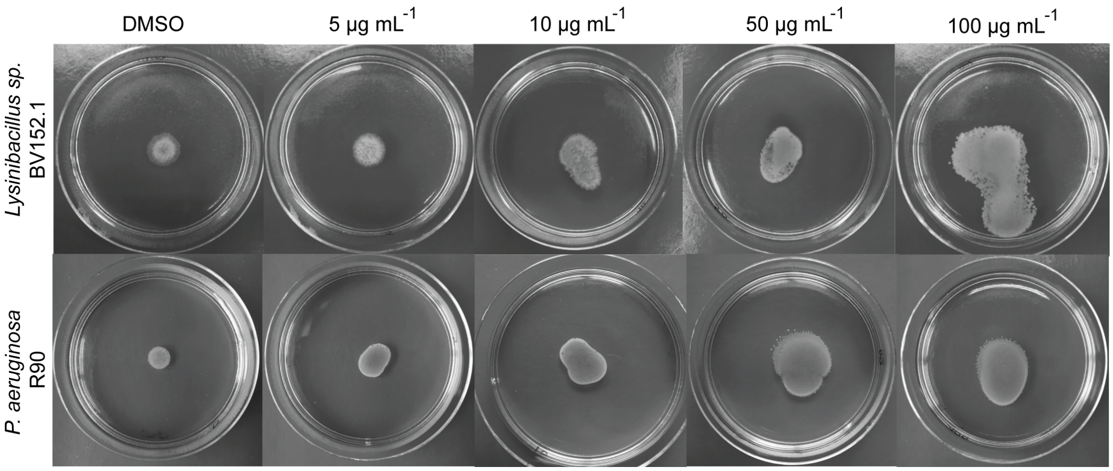

Supplementary Figure 1. Effects of rhamnolipid mixtures from *Lysinibacillus* sp. BV152.1 and *P. aeruginosa* on swarming activity in *P. aeruginosa* PAO1. Pictures were taken 24 h after inoculation.

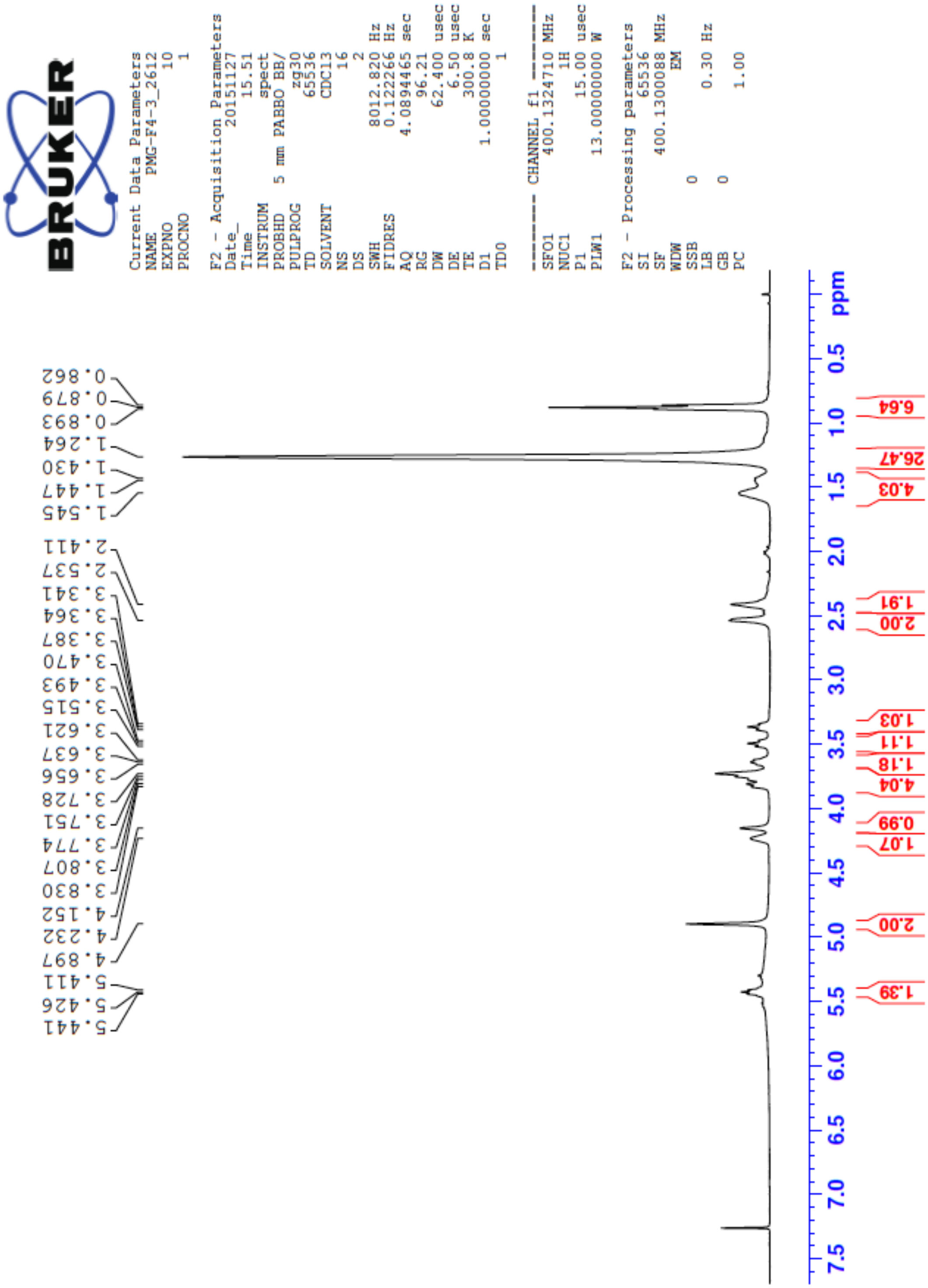

Supplementary Figure 2. NMR spectra ^{1}H of di-Rha.

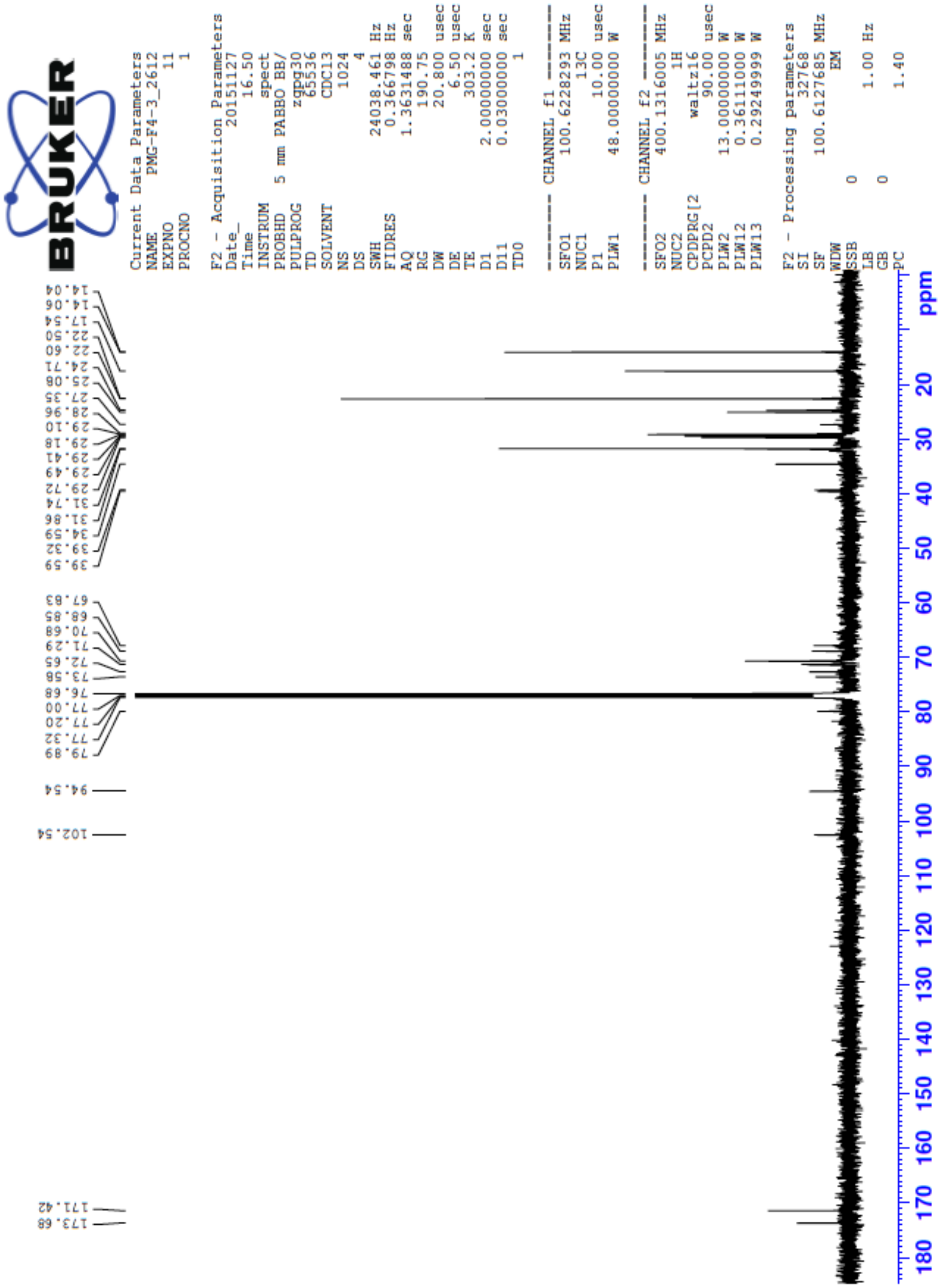

Supplementary Figure 3. NMR spectra ^{13}C of di-Rha.

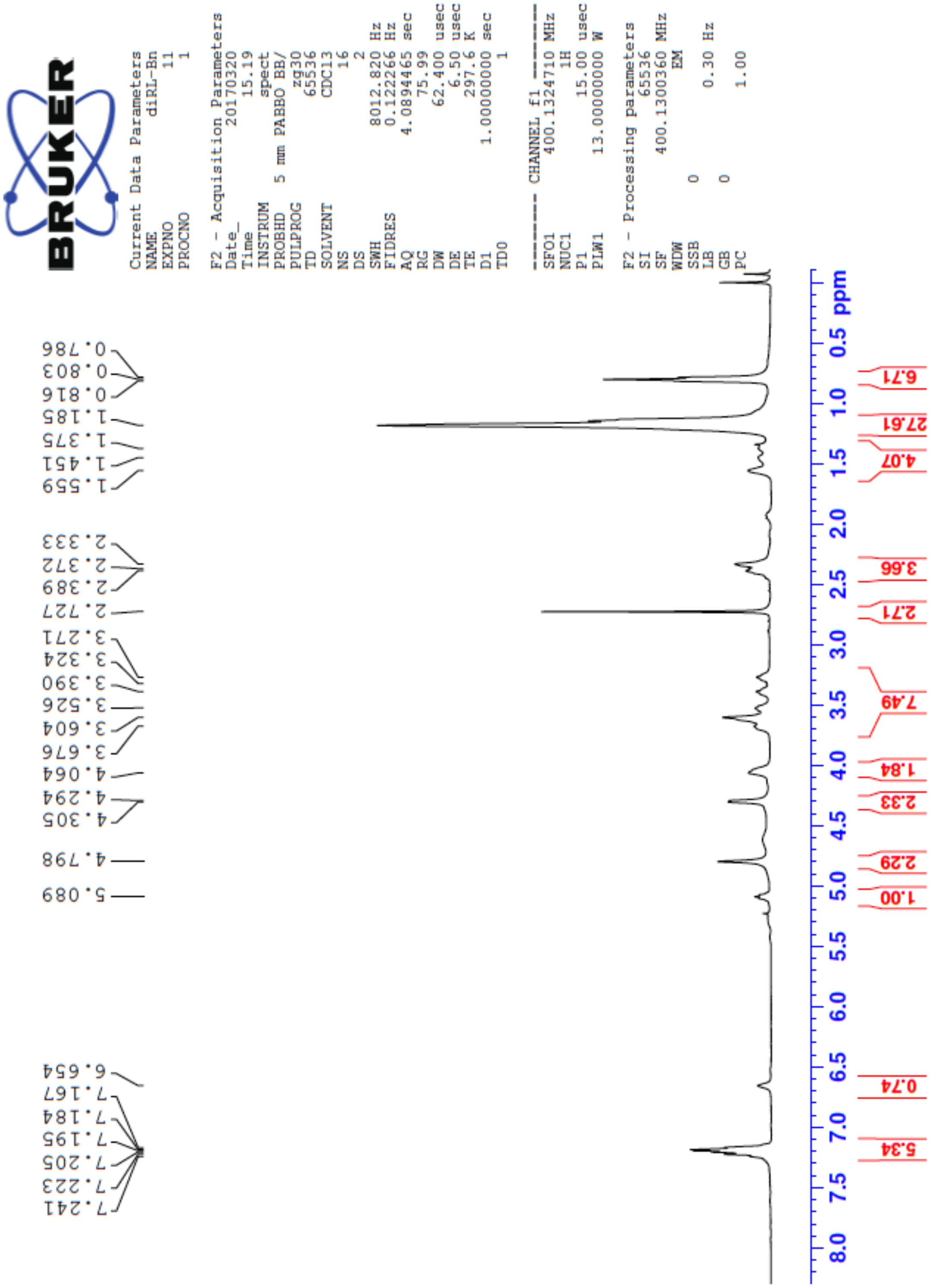

Supplementary Figure 4. NMR spectra ^{1}H of di-Rha-Bn.

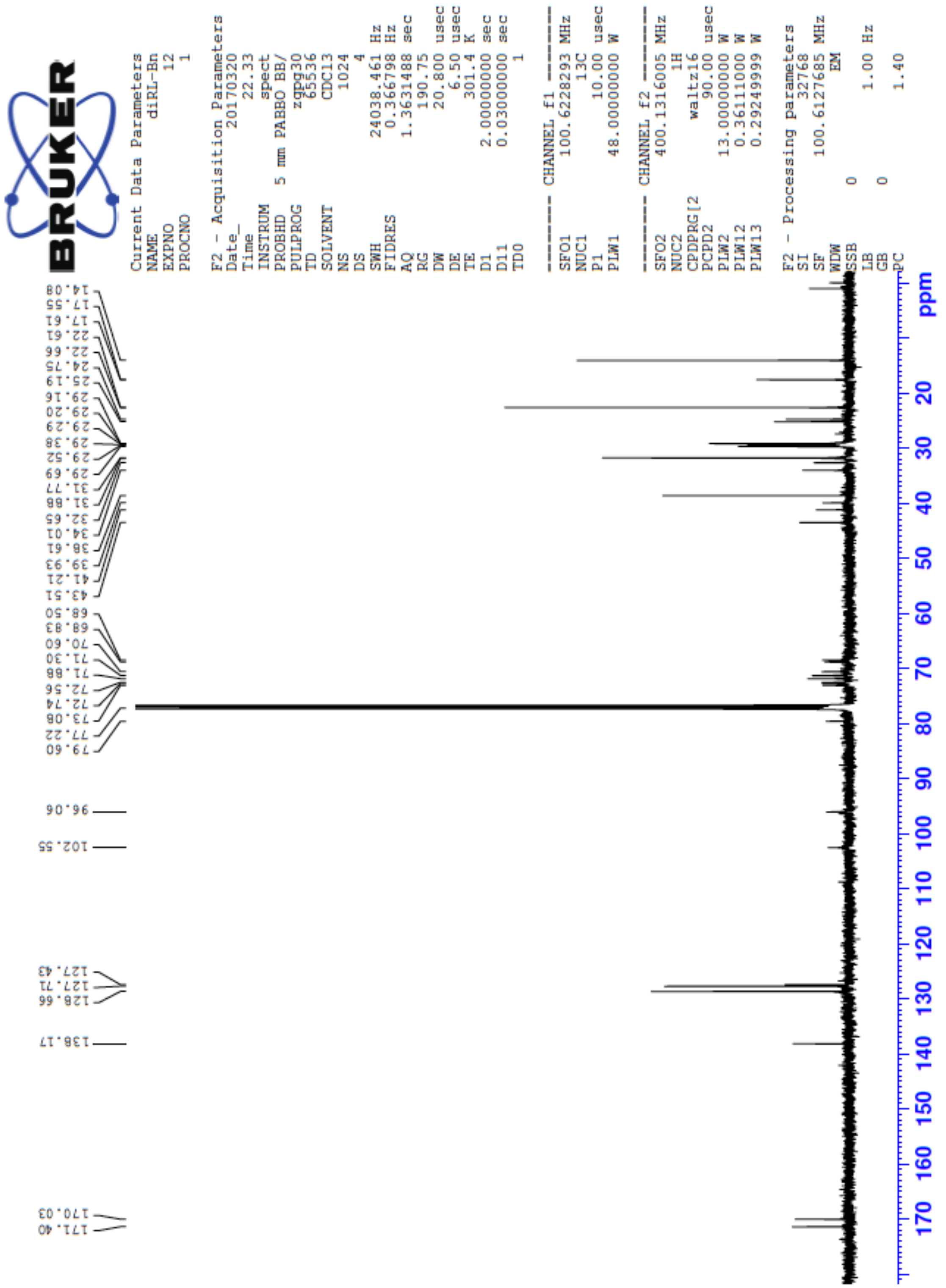

Supplementary Figure 5. NMR spectra ^{13}C of di-Rha-Bn.

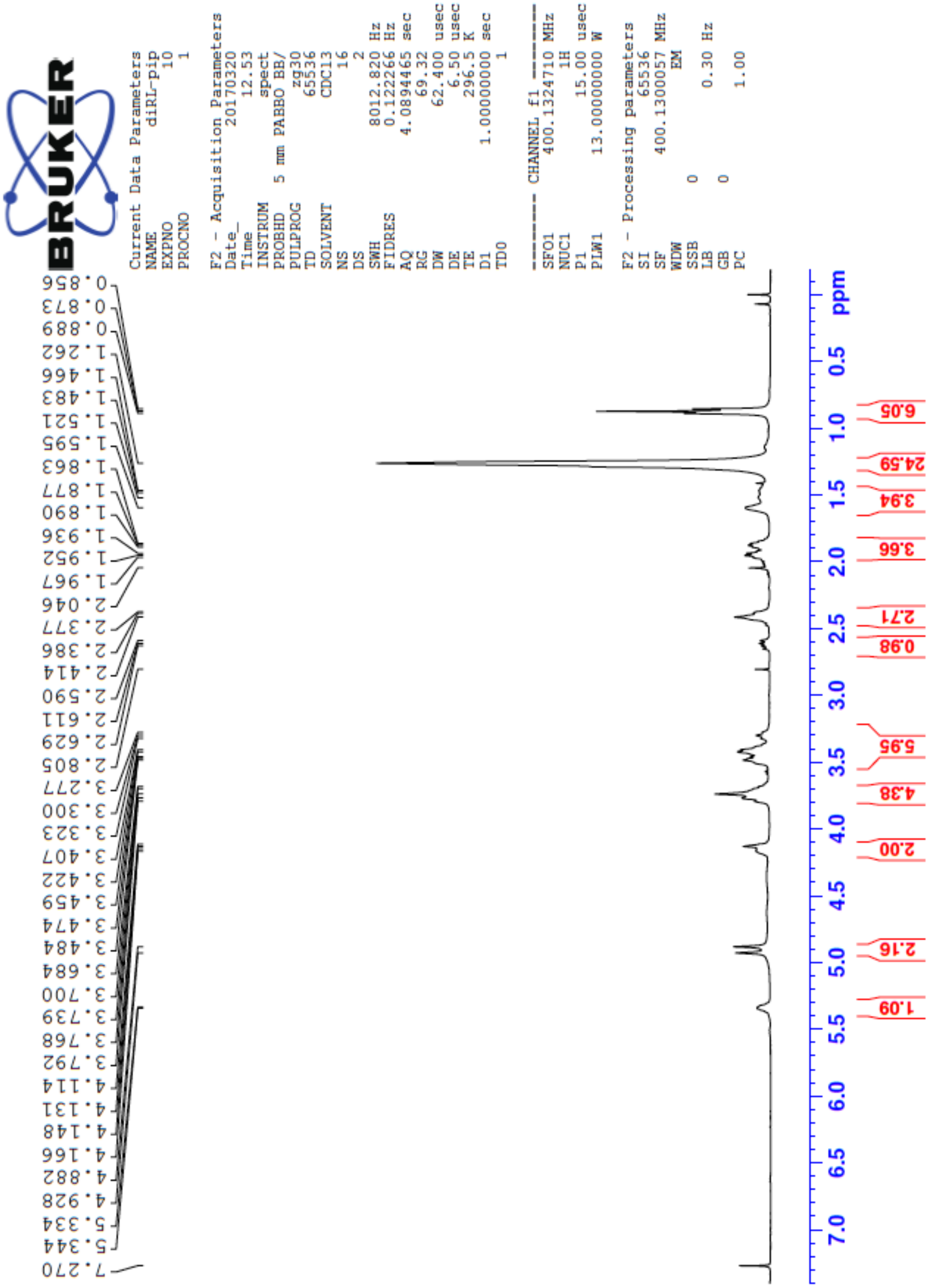

Supplementary Figure 6. NMR spectra ^{1}H of di-Rha-Pip.

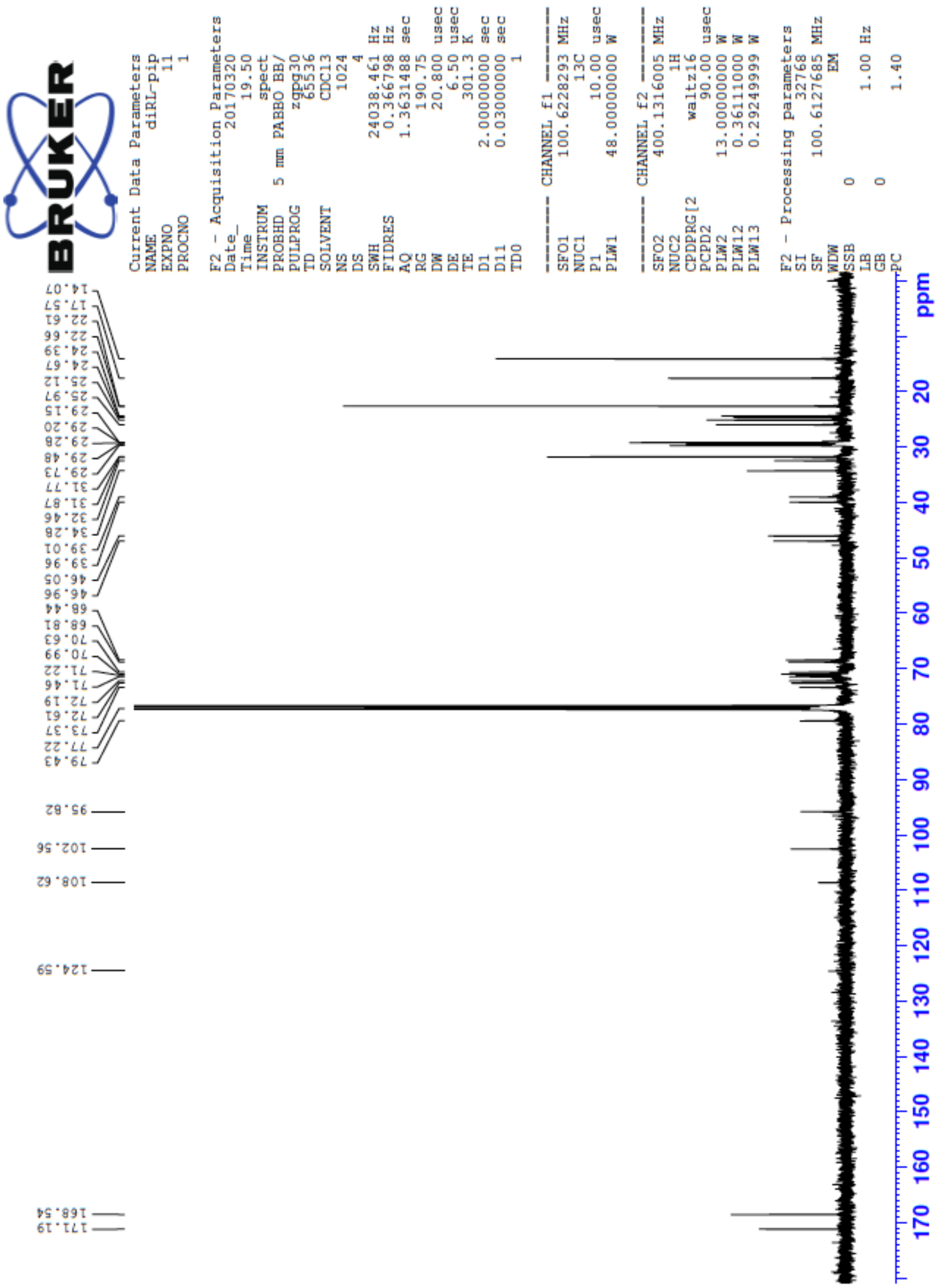

Supplementary Figure 7. NMR spectra ^{13}C of di-Rha-Pip.

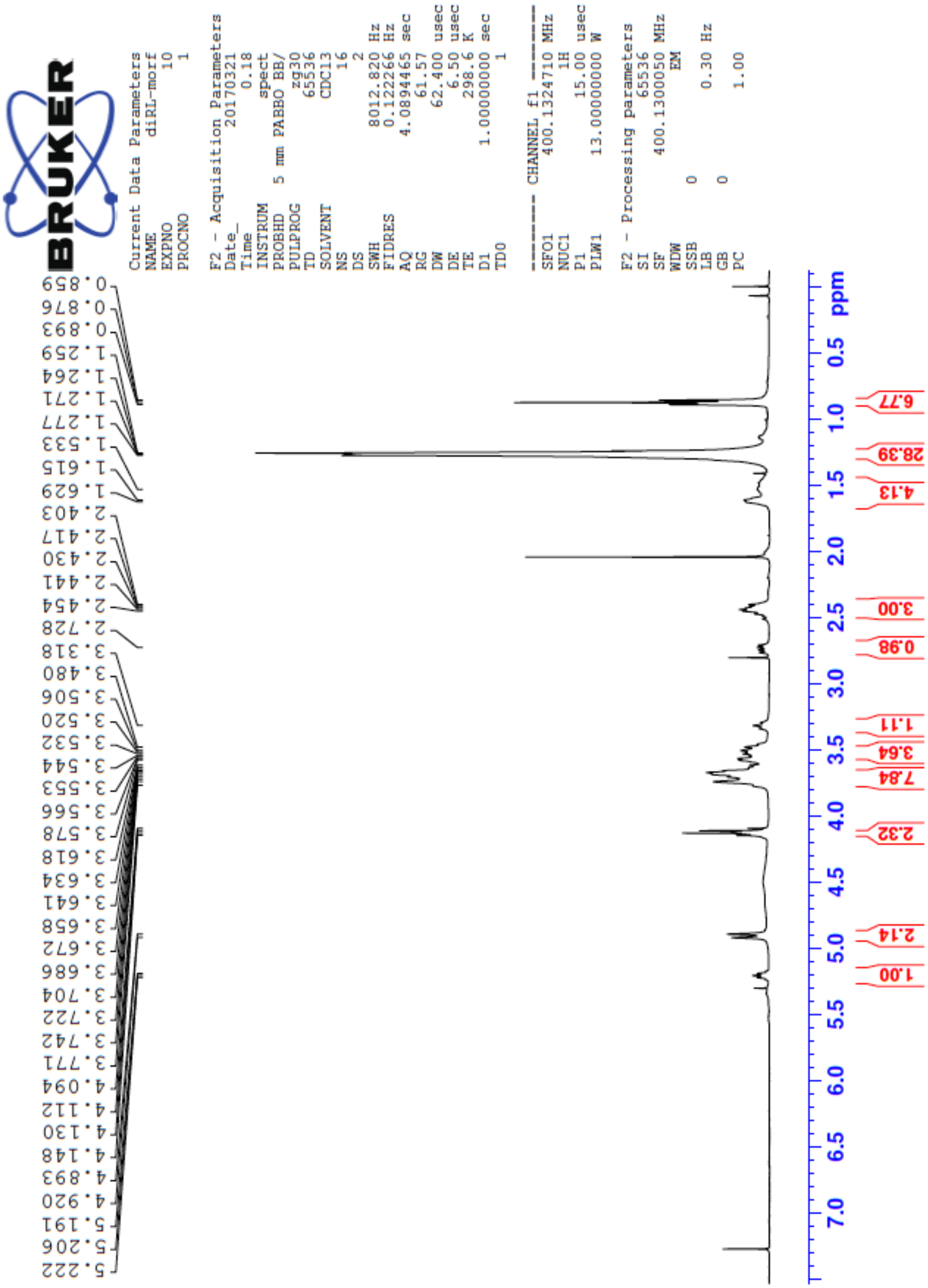

Supplementary Figure 8. NMR spectra ^{1}H of di-Rha-Mor.

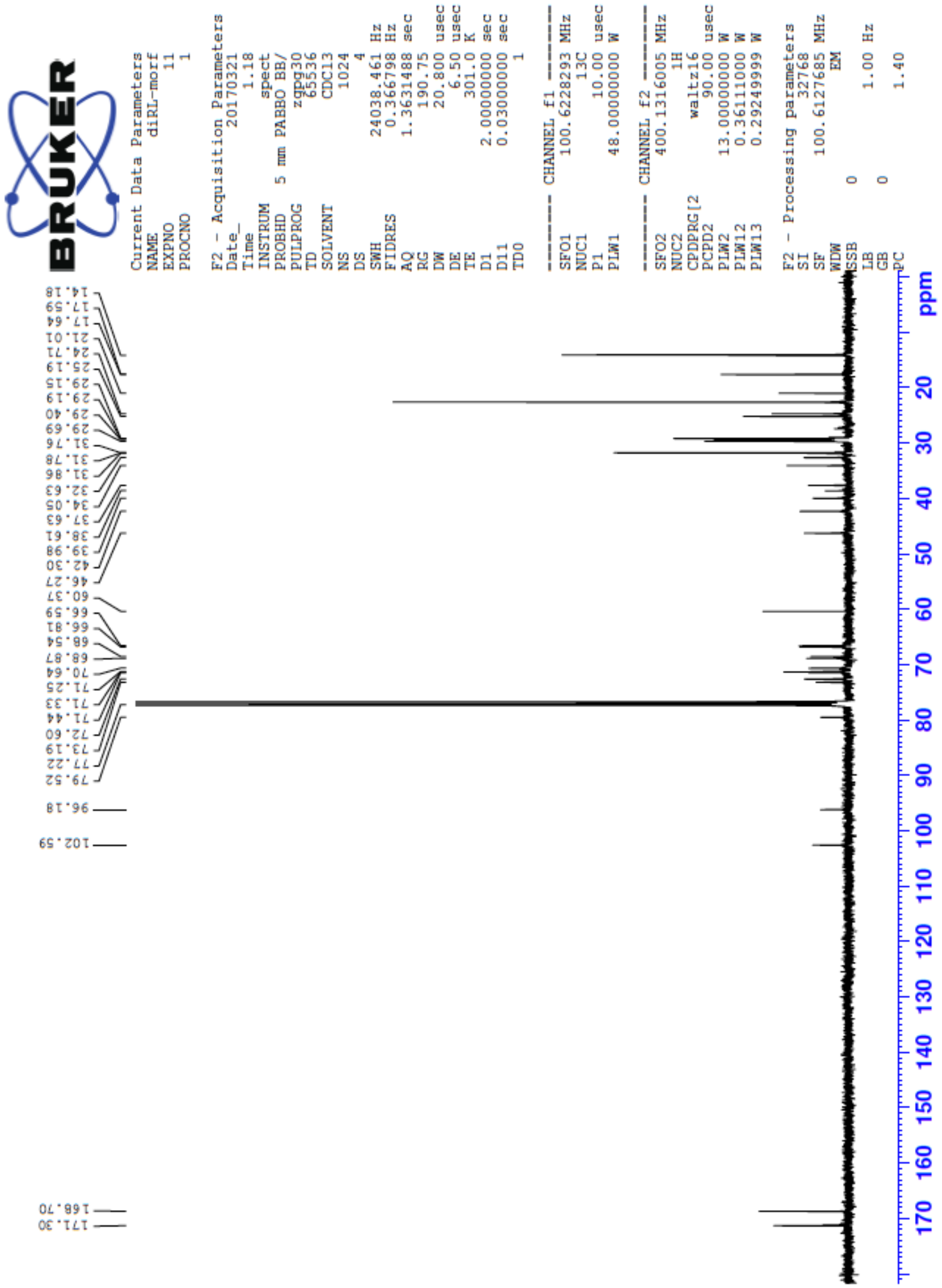

Supplementary Figure 9. NMR spectra ^{13}C of di-Rha-Mor.

Spectral data for the compounds:

F3 assigned spectral data are as follows: ^{1}H NMR (400 MHz, $CDCl_3$) δ 5.53 – 5.35 (m, 1H), 4.90 (s, 2H), 4.23 (s, 1H), 4.15 (s, 1H), 3.88 – 3.69 (m, 4H), 3.68 – 3.57 (m, 1H), 3.49 (t, *J* = 8.8 Hz, 1H), 3.36 (t, *J* = 9.2 Hz, 1H), 2.54 (s, 2H), 2.41 (s, 2H), 1.66 – 1.38 (m, 4H), 1.26 (s, 26H), 0.88 (t, *J* = 6.2 Hz, 6H).^{13}C NMR (101 MHz, $CDCl_3$) δ 173.7, 171.4, 102.5, 94.5, 79.9, 73.6, 72.7, 71.3, 70.9, 68.8, 67.8, 39.3, 34.6, 31.9, 31.7, 29.7, 29.4, 29.2, 29.1, 25.1, 24.7, 22.6, 17.5, 14.1, 14.0.

di-Rha-Bn (benzyl amide of di-rhamnolipids), 26% yield;

^{1}H NMR (400 MHz, $CDCl_3$) δ 7.24 – 7.17 (m, 5H), 6.65 (bs, 1H), 5.09 (bs, 1H), 4.79 (s, 2H), 4.30 (s, 2H), 4.06 (s, 2H), 3.67 – 3.27 (m, 7H), 2,72 (s, 2H), 2.38-2.33 (m, 4H), 1.56 – 1.37 (m, 4H), 1.18 (s, 26H), 0.80 (d, *J* = 6.6 Hz, 6H).

^{13}C NMR (101 MHz, $CDCl_3$) δ 171.4, 170.0, 138.2, 128.7, 127.7, 127.4, 102.6, 96.0, 79.6, 77.2, 73.1, 72.7, 72.6, 71.9, 71.3, 70.6, 68.8, 68.5, 43.5, 41.2, 39.9, 38.6, 34.0, 32.6, 31.9, 31.8, 29.7, 29.5, 29.4, 29.3, 29.2, 29.1, 25.2, 24.8, 22.7, 22.6, 17.6, 17.5, 14.1.

di-Rha-Pip (piperidine amide of di-rhamnolipids), 27% yield;

^{1}H NMR (400 MHz, $CDCl_3$) δ 5.34 (s, 1H), 4.92-4.88 (m, 2H), 4.16-4.11 (m, 2H), 3.79-3.68 (m, 4H), 3.48-3.27 (m, 6H), 2.61 (dd, *J* = 14.5, 7.5 Hz, 1H), 2.41 – 2.37 (m, 3H), 1.96-1.87 (m, 4H), 1.59-1.46 (m, 4H), 1.26 (s, 24H), 0.86 (d, *J* = 6.9 Hz, 6H).

^{13}C NMR (101 MHz, $CDCl_3$) δ 171.2, 168.5, 124.6, 108.6, 102.6, 95.8, 79.4, 77.2, 73.4, 72.6, 72.2, 71.5, 71.2, 71.0, 70.6, 68.8, 68.4, 47.0, 46.1, 40.0, 39.0, 34.3, 32.5, 31.9, 31.8, 29.7, 29.5, 29.3, 29.2, 29.1, 26.0, 25.1, 24.7, 24.4, 22.7, 22.6

di-Rha-Mor (morpholine amide of di-rhamnolipids), 25% yield;

^{1}H NMR (400 MHz, $CDCl_3$) δ 5.19 – 5.22 (m, 1H), 4.91 (d, *J* = 9.6 Hz, 2H), 4.14-4.09 (m, 2H), 3.77 – 3.48 (m, 10H), 3.32 (s, 1H), 2.73 (dd, *J* = 15.0, 7.2 Hz, 1H), 2.45 – 2.40 (m, 3H), 1.63 – 1.53 (m, 4H), 1.27 (s, 26H), 0.87 (t, *J* = 6.2 Hz, 6H).

^{13}C NMR (101 MHz, $CDCl_3$) δ 171.3, 168.7, 102.6, 96.2, 79.5, 77.2, 73.2, 72.6, 71.4, 71.3, 71.2, 70.6, 68.9, 68.6, 66.8, 60.4, 46.3, 42.3, 40.0, 38.6, 37.6, 34.0, 32.6, 31.9, 31.8, 29.7, 29.4, 29.2, 29.1, 25.2, 24.7, 21.0, 17.6, 17.5, 14.2, 14.1, 14.0

di-Rha-TBDMS (*t*-butyldimethylsilyl protected di-rhamnolipids), 55% yield

^{1}H NMR (400 MHz, $CDCl_3$) δ 5.40-5.17 (m, 1H), 4.87-4.48 (m, *2*H), 4.12-4.06 (m, *2*H), 3.97 – 3.19 (m, 7H), 2.54 – 2.17 (m, 4H), 1.66 – 1.25 (m, 8H), 1.25 – 1.01 (m, 32H), 0.87 – 0.61 (m, 45H), 0.21 – -0.31 (m, 26H).

^{13}C NMR (101 MHz, $CDCl_3$) δ 171.1, 167.7, 102.9, 102.5, 96.1, 82.2, 74.9, 73.3, 73.10, 71.7, 71.3, 70.9, 68.2, 39.8, 38.8, 34.9, 34.2, 33.1, 31.9, 31.7, 30.4, 30.3, 29.71, 29.5, 29.4, 29.2, 29.1, 28.9, 26.9, 26.4, 26.3, 26.01 25.9, 25.8, 25.7, 25.6, 25.2, 23.8, 23.0, 22.6, 18.3, 18.2, 18.1, 16.3, 14.1, 10.9, 1.0, -2.7, -2.9, -3.0, -3.3, -3.5, -3.7, -3.9, -4.3, -4.4, -4.6, -4.7.

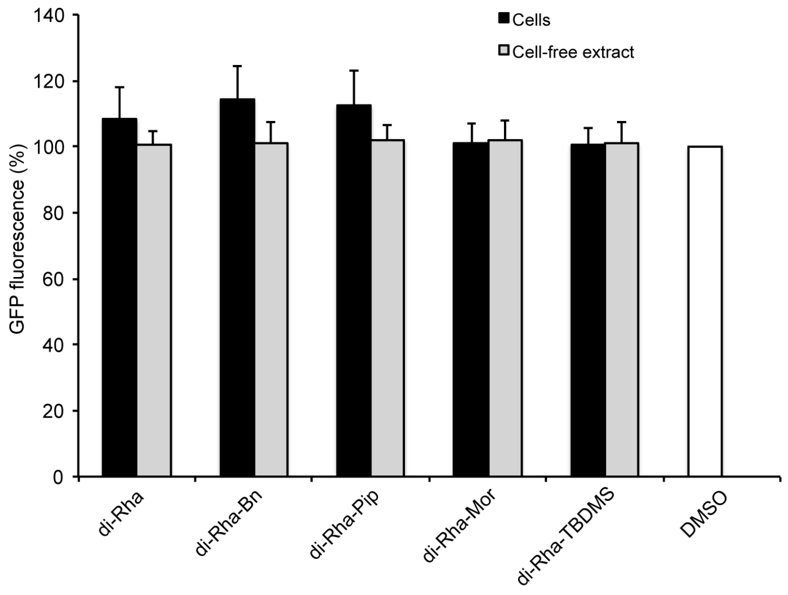

Supplementary Figure 10. Effects of di-rhamnolipids and their derivatives on GFP expression and its fluorescence. GFP fluorescence in bacterial cells was expressed as fluorescence/ OD_{600}.

Supplementary references

da Silva, D.P., Castaneda-Ojeda, M.P., Moretti, C., Buonaurio, R., Ramos, C., and Venturi, V. 2014. Bacterial multispecies studies and microbiome analysis of a plant disease. Microbiology-Sgm 160, 556-566.

Ha, D.G., Kuchma, S.L., and O'Toole, G.A. 2014. Plate-based assay for swarming motility in *Pseudomonas aeruginosa*. Methods Mol Biol 1149, 67-72.
